# From strain engineering to process development: monoclonal antibody production with an unnatural amino acid in *Pichia pastoris*

**DOI:** 10.1186/s12934-022-01882-6

**Published:** 2022-08-11

**Authors:** Nora Tir, Lina Heistinger, Clemens Grünwald-Gruber, Leo A. Jakob, Stephan Dickgiesser, Nicolas Rasche, Diethard Mattanovich

**Affiliations:** 1 Christian Doppler Laboratory for Innovative Immunotherapeutics, Department of Biotechnology, University of Natural Resources and Life Sciences, Muthgasse 18, 1190 Vienna, Austria; 2 Department of Biotechnology, Institute of Microbiology and Microbial Biotechnology, University of Natural Resources and Life Sciences, Muthgasse 18, 1190 Vienna, Austria; 3 University of Natural Resources and Life Sciences, Vienna Core Facility Mass Spectrometry, Muthgasse 18, 1190 Vienna, Austria; 4 Department of Biotechnology, Institute of Bioprocess Science and Engineering, University of Natural Resources and Life Sciences, Muthgasse 18, 1190 Vienna, Austria; 5 ADCs & Targeted NBE Therapeutics, Merck Healthcare KGaA, Frankfurter Str. 250, 64293 Darmstadt, Germany; 6 Present Address: Department of Biology, Institute of Biochemistry, ETH Zürich, 8093 Zurich, Switzerland

**Keywords:** *Pichia pastoris*, Monoclonal antibody, Unnatural amino acid, Strain engineering, Bioreactor cultivation

## Abstract

**Background:**

Expansion of the genetic code is a frequently employed approach for the modification of recombinant protein properties. It involves reassignment of a codon to another, e.g., unnatural, amino acid and requires the action of a pair of orthogonal tRNA and aminoacyl tRNA synthetase modified to recognize only the desired amino acid. This approach was applied for the production of trastuzumab IgG carrying *p*-azido-l-phenylalanine (pAzF) in the industrial yeast *Pichia pastoris*. Combining the knowledge of protein folding and secretion with bioreactor cultivations, the aim of the work was to make the production of monoclonal antibodies with an expanded genetic code cost-effective on a laboratory scale.

**Results:**

Co-translational transport of proteins into the endoplasmic reticulum through secretion signal prepeptide change and overexpression of lumenal chaperones Kar2p and Lhs1p improved the production of trastuzumab IgG and its Fab fragment with incorporated pAzF. In the case of Fab, a knockout of vacuolar targeting for protein degradation further increased protein yield. Fed-batch bioreactor cultivations of engineered *P. pastoris* strains increased IgG and IgG_pAzF_ productivity by around 50- and 20-fold compared to screenings, yielding up to 238 mg L^−1^ and 15 mg L^−1^ of fully assembled tetrameric protein, respectively. Successful site-specific incorporation of pAzF was confirmed by mass spectrometry.

**Conclusions:**

*Pichia pastoris* was successfully employed for cost-effective laboratory-scale production of a monoclonal antibody with an unnatural amino acid. Applying the results of this work in glycoengineered strains, and taking further steps in process development opens great possibilities for utilizing *P. pastoris* in the development of antibodies for subsequent conjugations with, e.g., bioactive payloads.

**Supplementary Information:**

The online version contains supplementary material available at 10.1186/s12934-022-01882-6.

## Background

In the past two decades, the demand for site-specific incorporation of reactive chemical groups in a wide range of proteins has been growing to gain novel properties. Particularly after the formulation of the official definition of “click” chemistry [1], research has focused on the incorporation of unnatural amino acids (uAAs) into proteins with reactive groups that cannot be found in nature and react with biological systems. Such reactions are bioorthogonal and, frequently, they proceed in one step, without the formation of side products. These desirable features have opened doors to various applications and manipulations of endogenous and heterologous proteins [2, 3].

Several conditions need to be fulfilled to successfully incorporate an uAA at the desired position in a target protein. The uAA to be site-specifically incorporated must not be recognized by endogenous tRNAs and tRNA synthetases (RSs). In other words, the technology used and the uAA must be orthogonal in the host cell. The most common approach to achieving orthogonality is using a tRNA and its cognate RS from an organism phylogenetically distant from the expression host. Altogether, the codon reassignment by which an uAA is site-specifically incorporated instead of a canonical amino acid is called the expansion of the genetic code. It is frequently employed for the incorporation of uAAs into monoclonal antibodies and antibody formats, predominantly of the IgG1 subclass, to enable site-specific click reactions with small molecule payloads. The resulting products are known as antibody-drug conjugates (ADCs) and extend the usage of immunotherapeutics. An overview of Food and Drug Administration-approved ADCs currently on the market is given by Tong et al. [4].

The growing need for fast and cost-effective antibody development and production requires finding suitable expression hosts. Unlike the production of antibody fragments, for which bacterial systems can be used, tetrameric IgGs with suitable glycosylation patterns require eukaryotic hosts. The simplest and inexpensive eukaryotic hosts are yeasts that have already shown the potential for full-length IgG production [5, 6]. By combining glycoengineering [7], optimization of protein folding and secretion [8, 9], and process development [10, 11], yeast research is gaining more attention for the fast and cheap production of recombinant therapeutics. The industrial yeast *Pichia pastoris* (syn. *Komagataella* spp.) is one of the well-established microbial hosts for recombinant protein production. Like other yeast species, it requires inexpensive media for growth, can produce proteins with post-translational modifications including human-type glycosylation [12], and has a higher secretion capacity than prokaryotes. Moreover, *P. pastoris*, unlike *Saccharomyces cerevisiae*, has a Crabtree-negative phenotype [13, 14], facilitating its cultivation at high cell densities to obtain higher product titers. Due to its methylotrophic metabolism and genes tightly regulated by methanol, it is commonly used for the inducible production of large amounts of heterologous proteins [15]. In addition, *P. pastoris* secretes very few endogenous proteins in the supernatant, which reduces the effort to purify the secreted product.

Research on (heterologous) protein folding and secretion in *P. pastoris* has advanced significantly in the last decade. A growing need for cost-effective production of recombinant therapeutics and industrial enzymes pushes the need for strain and metabolic engineering of this yeast. Protein folding and secretion pathways in *P. pastoris* were discussed in detail by Damasceno et al. [16] and Delic et al. [17]. Furthermore, successful incorporations of uAAs into recombinant proteins produced by *P. pastoris* were previously reported [18, 19]. In the present study, we combined our knowledge of *P. pastoris* with the ongoing requirements for improving monoclonal antibody production with an expanded genetic code to relieve some of the major bottlenecks in the secretion of trastuzumab antigen-binding fragment (Fab) and full-length IgG with site-specifically incorporated *p*-l-azidophenylalanine (pAzF).

## Results

### Influence of tRNA_CUA_/RS expression on pAzF incorporation yield

Genes for the orthogonal pair of amber suppressor tRNA (tRNA_CUA_) and pAzF-specific RS (RS^pAzF^) were derived from *E. coli* tyrosyl-tRNA_CUA_ and tyrosyl-RS, as reported previously [18, 20, 21]. Methanol-inducible promoters of varying strength were chosen for each gene based on the knowledge of the methanol utilization pathway in *P. pastoris* [15, 22]. Enhanced green fluorescent protein (eGFP) was used as a reporter protein for pAzF incorporation in small-scale screenings and detection was done by flow cytometry. The codon for tyrosine-40 in the sequence was replaced with the amber stop codon (eGFP_Y40X_). For eGFP expression, the strong methanol-inducible promoter for alcohol oxidase 1 was used. All cloning was performed using Golden*Pi*CS [23] and the corresponding schemes are shown in Additional file 1: Fig. S1.

**Fig. 1:**
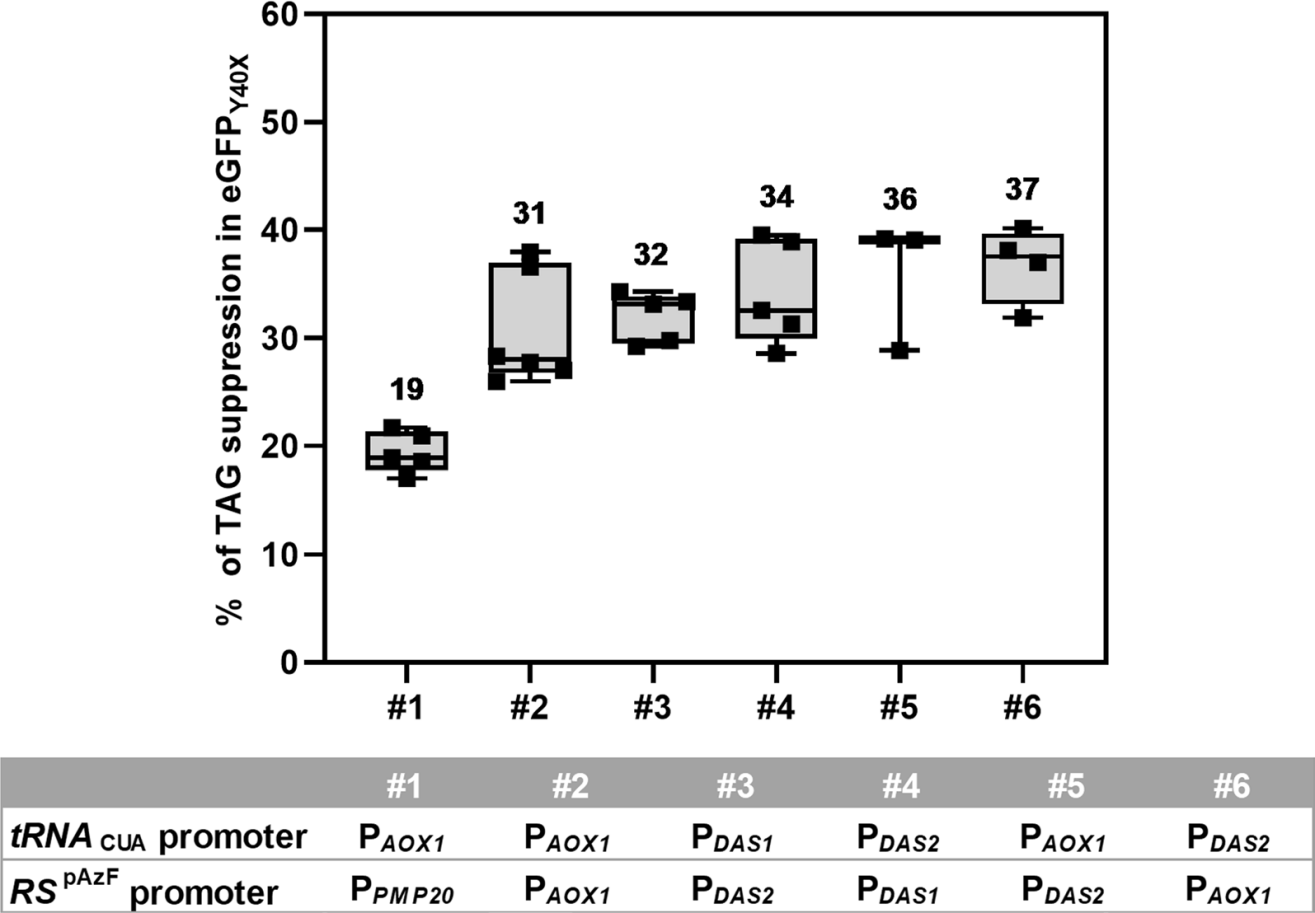
Amber suppression in eGFP_Y40pAzF_ depends on the promoter combination in the tRNA_CUA_/RS^pAzF^ pair. Mean values above the boxes are percentages calculated from the relative amounts of eGFP_Y40pAzF_ to eGFP produced per cell from three to eight biological replicates and are shown as horizontal lines in the boxes. Whiskers represent minimal and maximal values. The table below the graph shows the identity of the promoter combinations

Promoter strengths in the presence of methanol were previously determined by microarray analysis and decreases in the following order [22]: dihydroxyacetone synthase 2 (P_*DAS2*_) > alcohol oxidase 1 (P_*AOX1*_) > dihydroxyacetone synthase 1 (P_*DAS1*_) > peroxisomal membrane-associated protein 20 (P_*PMP20*_), where the relative P_*PMP20*_ strength has not been published yet. Although less than 5% of the overall difference in stop codon suppression was observed between the cultures expressing both tRNA_CUA_ and RS^pAzF^ under the strongest methanol-inducible promoters in *P. pastoris*, it was evident that a strong expression was required for tRNA_CUA_ (Fig. 1). It has already been established that increasing the tRNA_CUA_ gene copy number above 3 does not improve the stop codon suppression and that the addition of a polymerase II promoter in front of the polymerase III promoter further increases expression levels [21]. In this work, the construct with 3 tandem copies of tRNA_CUA_, each lacking the CCA trinucleotide at its 3′ terminus, flanked by the *SUP4* region from *S. cerevisiae* was used. In addition, the choice of different methanol-inducible promoters for each gene placed in the same vector further increases genome stability when prolonged cultivation times are needed. Therefore, the combination of the P_*DAS2*_ for tRNA_CUA_ (P_*DAS2*__3x*SUP4*-tRNA_CUA_) and P_*AOX1*_ for RS (P_*AOX1*__*RS*^pAzF^) was used in all other experiments. The optimum pAzF concentration in the culture medium was determined as 2 mM, leading to the maximum suppression with the least possible impact on biomass growth (Additional file 2: Table S1).

### Tuning light and heavy chain expression

**Fig. 2:**
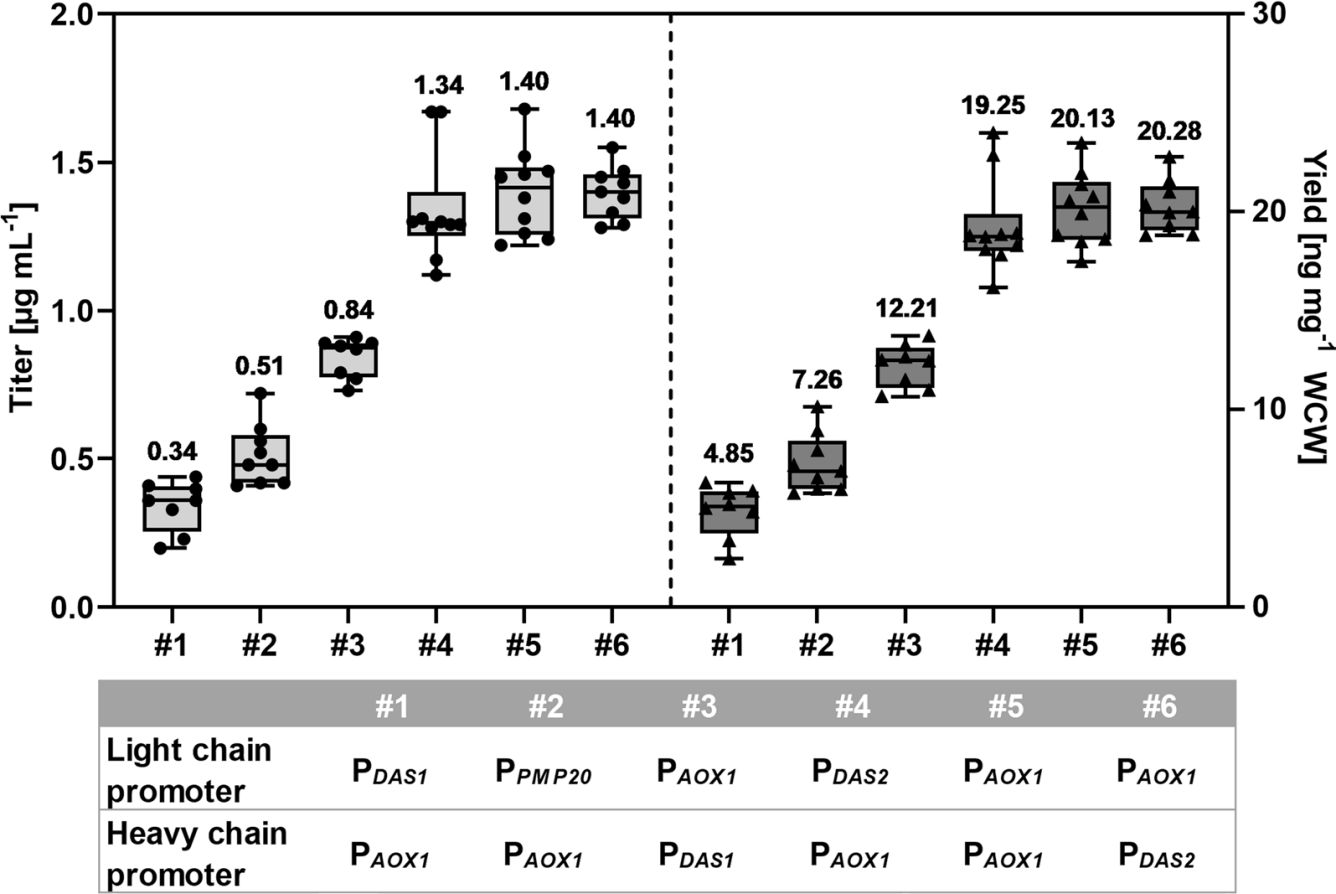
Titers and yields of the wild-type trastuzumab Fab depend on the promoter combination. Mean values above the boxes were calculated from seven to ten biological replicates and are shown as horizontal lines. Dots and triangles show each data point and whiskers show minimal and maximal values. The yields were calculated as the amount of protein per wet cell weight (WCW). The table below the graph shows the identity of the promoter combinations

Monoclonal antibody production in microbial hosts is challenging and product-dependent. In shake-flask cultivations, most of the secreted product is reduced antibody formats rather than fully-assembled tetrameric antibodies [6]. In order to monitor product quality and quantity, *P. pastoris* secreting trastuzumab Fab fragment fused to the prepeptide for oligosaccharide transferase 1 and the propeptide for mating factor alpha of *S. cerevisiae* (pre-Ost1-pro-MFα) was used [24, 25]. In the screenings for Fab expression, different methanol-inducible promoters for each chain were tested (Fig. 2). When the same choice of promoters was used in the opposite order, the combination with a stronger promoter for the light chain resulted in more than a twofold higher product titer and yield, supporting the previous observation by Gasser et al. [8]. However, overall strong expression of both chains was required for the best product titer and yield. Therefore, the combination used in all further experiments with Fab and IgG was P_*AOX1*_ for the light chain (P_*AOX1*__*LC*) and P_*DAS2*_ for the heavy chain (P_*DAS2*__*HC*).

### Changing the secretion signal prepeptide to improve protein secretion

**Fig. 3:**
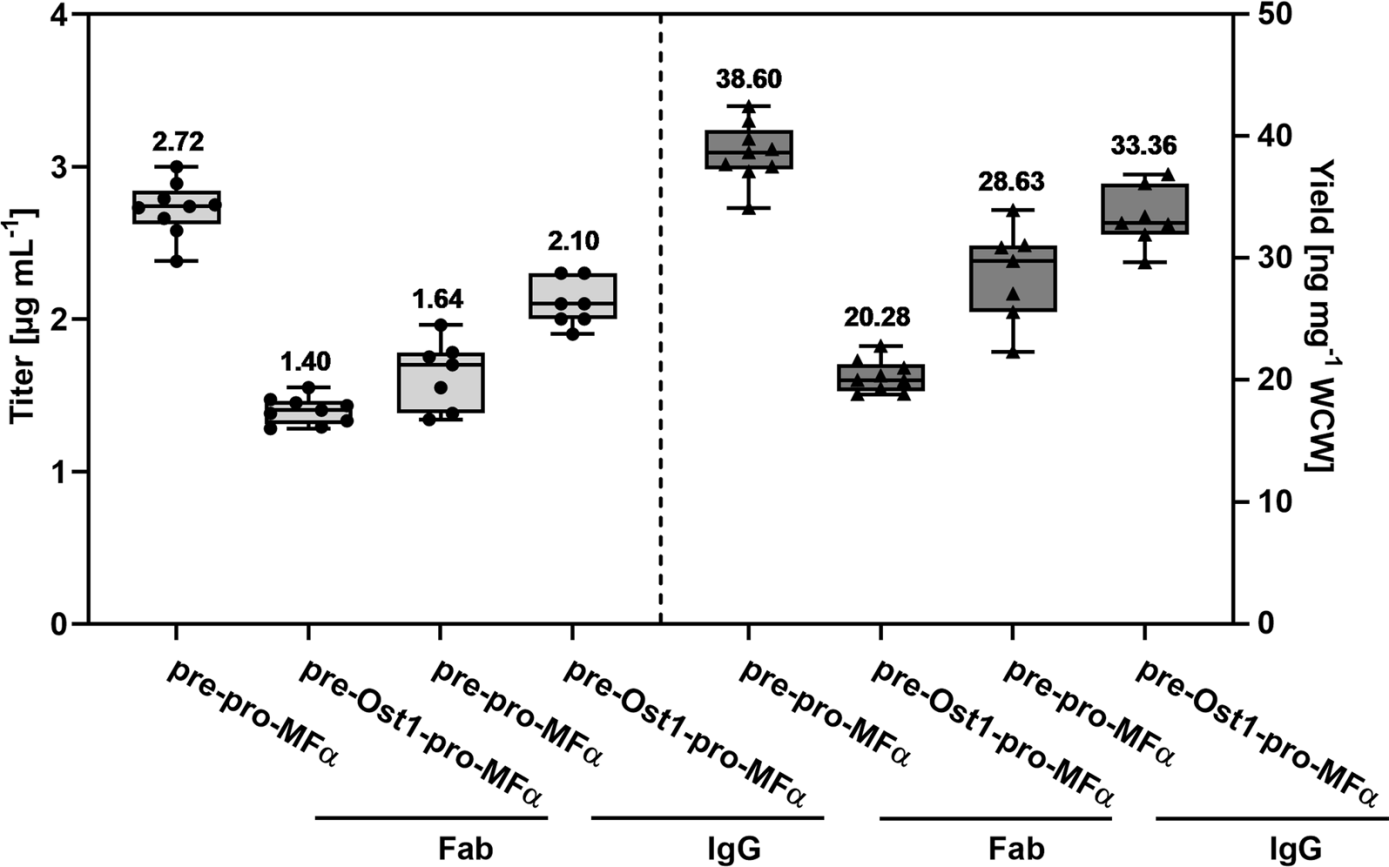
Influence of secretion signals on Fab and IgG titers (left) and yields (right). Mean values above the boxes were calculated from seven to nine biological replicates and are shown as horizontal lines. Dots and triangles show each data point and whiskers show minimal and maximal values. The yields were calculated as the amount of protein per wet cell weight (WCW)

Aside from promoter selection, the effects of the pre-Ost1-pro-MFα secretion signal and the standard pre-pro-secretion signal for the mating factor α (pre-pro-MFα) on the secretion of both trastuzumab Fab and IgG were compared. The mean titer and yield of Fab fused to the pre-pro-MFα were almost twofold higher than that of Fab fused to the pre-Ost1-pro-MFα secretion signal (Fig. 3). In the case of IgG, titer and yield of pre-Ost1-pro-MFα fused protein were about 1.3-fold and 1.1-fold higher than those of pre-pro-MFα fused one, respectively. However, when examining supernatants from the screenings by SDS-PAGE and western blot (Additional file 1: Fig. S2), significantly more endogenous proteins were observed in the cultures producing Fab and IgG fused to the pre-pro-MFα secretion signal. Since protein purification from bioreactor cultivations was the final goal, the pre-Ost1-pro-MFα secretion signal was chosen for all other experiments.

Additionally, no band corresponding to the tetrameric protein (HC_2_-LC_2_) was observed in both cases. On the screening scale, this is not unusual and depends on the exact identity of IgG. Nevertheless, it was still possible to detect protein by ELISA because the antibody combination used binds the heavy chain and detects any species with assembled Fab domain, namely HC-LC, HC_2_-LC, and, if present, HC_2_-LC_2_.

### Strain improvement by helper factor overexpression

The choice of targets for overexpression and knockout in this work was based on the previous experience with Fab and, to a certain extent, IgG production in yeast. The list with the target description and references is given in Table 1.

**Table 1:** Overview of overexpression and knockout targets tested in this work for IgG producing *P. pastoris*

Gene	Name description	Product description	Cellular localization	References
*YAP1*	Yeast activator protein 1	Transcription factor involved in the regulation of transcription in response to oxidative stress	Nucleus and cytoplasm	[26]
*HAC1*	Homologous to Atf/Creb1	Transcription factor involved in the regulation of the unfolded protein response	Nucleus	[27]
*PDI1*	Protein disulfide isomerase 1	Multifunctional protein disulfide isomerase and protein disulfide oxidoreductase	ER lumen	[8]
*SBH1*	Sec61 beta homolog 1	Beta subunit of Sec61 ER translocation complex	ER membrane	[28]
*CPR5*	Cyclosporin-sensitive proline rotamase 5	Rearranges peptidyl-prolyl bonds; transcriptionally induced in response to the UPR	ER lumen	[9]
*KAR2*	Karyogamy gene 2	ATPase involved in protein import into the ER, mediates protein folding, and regulates UPR	ER lumen	[6]
*LHS1*	Luminal Hsp70 protein 1	UPR regulated chaperone of the ER lumen involved in polypeptide translocation and folding	ER lumen	[29]
*YPT7*^a^	Yeast protein transport 7	Rab family GTPase involved in membrane trafficking	Vacuolar, endosomal, Golgi and mitochondrial membrane	[28]

^a^Knockout target

**Fig. 4:**
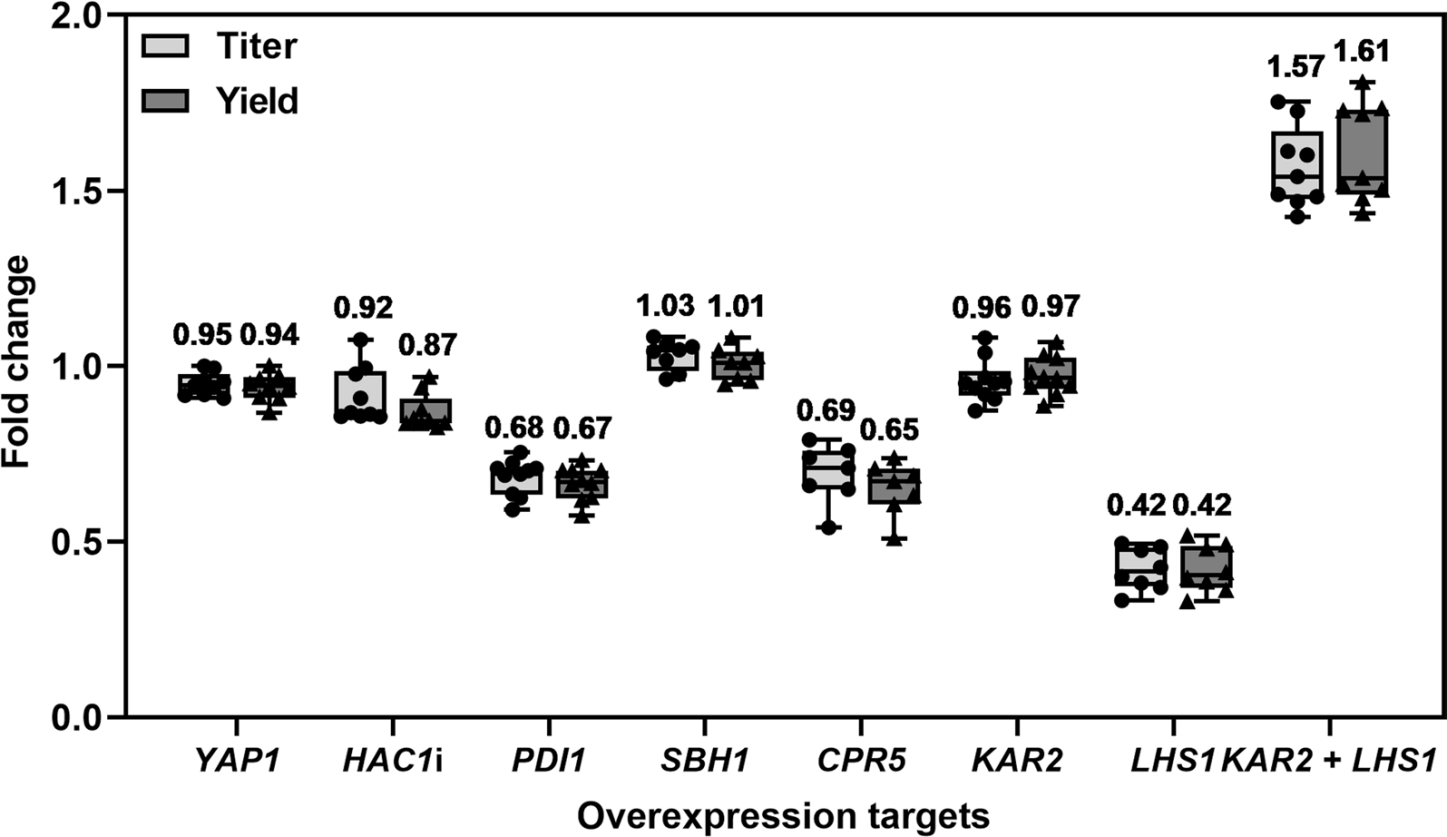
Influence of helper factor overexpression on IgG production. Titers are shown in light grey boxes and yields in dark grey boxes. Mean values above the boxes were calculated from seven to ten biological replicates (dots and triangles) relative to the parent strain without overexpression and are shown as the horizontal line in each box. Whiskers show minimal and maximal values

Overexpression of the transcription factors *HAC1*i (intronless *HAC1*) and *YAP1* did not improve IgG titer and yield (Fig. 4). Overexpression of *PDI1* reduced IgG titer and yield by 30%, in contrast to some of the reports for recombinant protein production in *P. pastoris* [8, 27]. Together with the lack of improvement of IgG secretion upon *HAC1*i and *YAP1* overexpression, this observation points towards other bottlenecks of *P. pastoris* as a monoclonal antibody production host.

Sbh1p is a subunit of the Sec61p ER translocation complex (in addition to Sec62p, Sec63p, Sec71p, and Sec72p) that interacts with the exocyst and the ribosome during co-translational translocation into the ER. It was expected that *SBH1* overexpression has a positive effect on IgG secretion. However, no change in IgG titer and yield was observed in this work.

A positive impact of overexpression of the peptidyl-prolyl isomerase *CPR5* in *S. cerevisiae* producing a full-length human IgG was reported [9]. Therefore, it appeared as a promising target for improving IgG production in *P. pastoris*. Nevertheless, a reduction of 30% in both IgG titer and yield was observed upon *CPR5* overexpression. As with *PDI1* overexpression, the observed effect could be a consequence of the protein burden in the ER or protein misfolding due to the constitutive *CPR5* expression.

Kar2p is the ER chaperone of the Hsp70 family whose overexpression generally increases recombinant protein secretion. Similar to the observation in *S. cerevisiae* [9], *KAR2* overexpression under the glyceraldehyde-3-phosphatase promoter (P_*GAP*_) did not improve IgG titer and yield. Lhs1p functions as a nucleotide exchange factor (NEF) that interacts with Kar2p [30]. Reduced IgG titer and yield by 60% were observed upon *LHS1* overexpression under the porine 1 promoter (P_*POR1*_), which has 50% strength compared to P_*GAP*_ [23]. This is likely due to the chaperone disbalance in the ER, although positive effects were observed upon *LHS1* overexpression in *S. cerevisiae* producing recombinant human albumin [29]. In this work, the balance between Kar2p and Lhs1p activity was crucial for the production of both Fab (not shown) and IgG. Co-overexpression of the two ER chaperones increased IgG titer and yield around 1.6-fold, confirming the previous observation that the bottleneck in *P. pastoris* producing Fab is the ER import [31]. The finding was then applied to the strains producing the IgG variant carrying pAzF in the light chain at position Y173 and compared with Fab-producing analogs.

### Disruption of vacuolar targeting further increased recombinant protein secretion

**Fig. 5:**
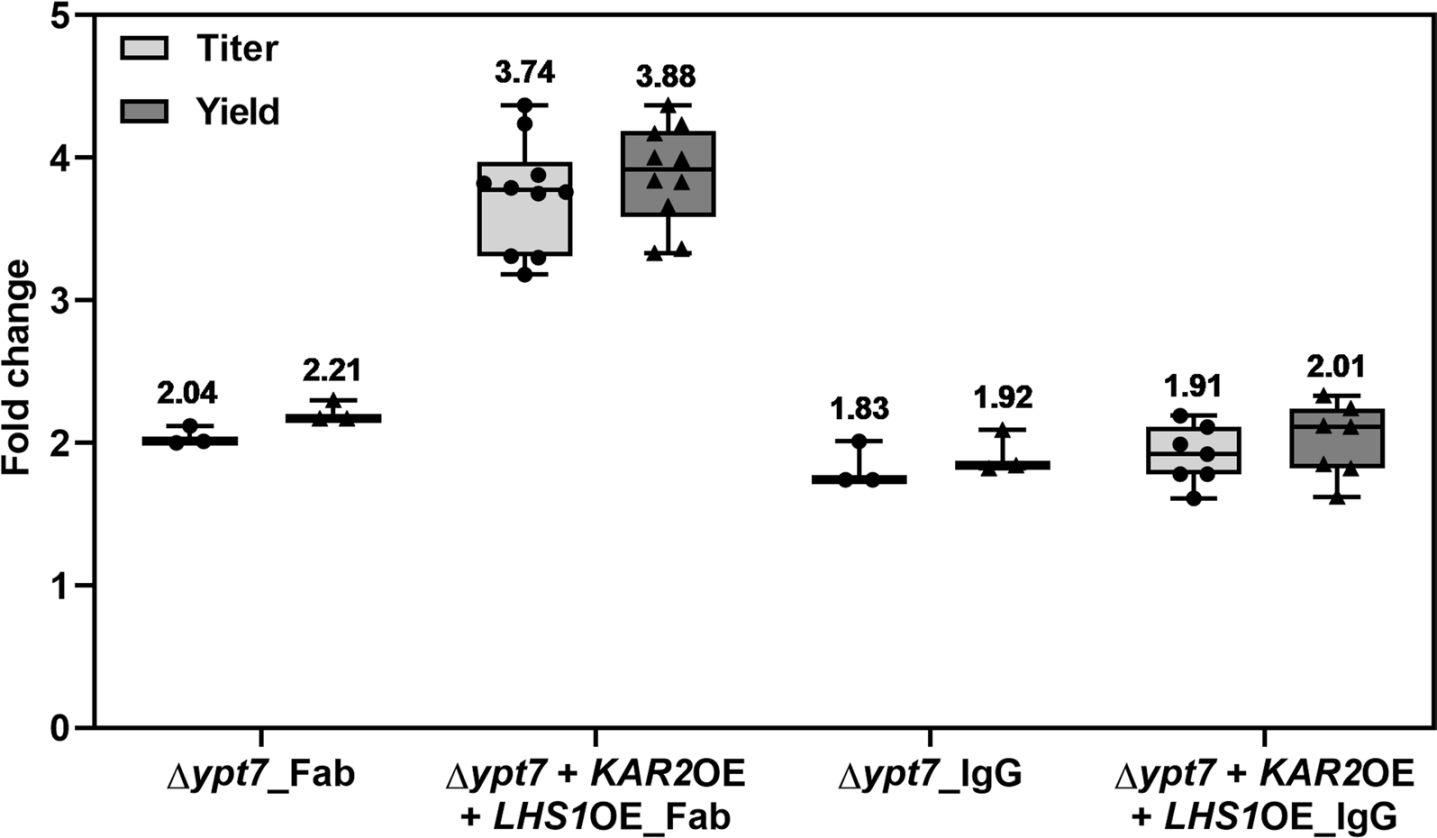
Influence of *YPT7* disruption and *KAR2* and *LHS1* co-overexpression on Fab and IgG production. Titer fold changes are shown in light grey boxes and yield fold changes in dark grey boxes. Mean values above the boxes were calculated from three to ten biological replicates (dots and triangles) relative to the wild-type strain expressing either Fab or IgG and are shown as the horizontal line in each box. Whiskers show minimal and maximal values

An additional bottleneck in IgG secretion was tackled by deleting the gene for Ypt7p, a Rab-family GTPase involved in membrane trafficking and mediates endosome-to-vacuole transport for protein degradation [32]. *YPT7* deletion strains producing Fab and IgG were generated using the CRISPR/Cas9 approach with a template DNA containing *YPT7* flanking regions. Fab titer was increased by around twofold, and IgG titer by around 1.8-fold upon *YPT7* deletion (Fig. 5). The additive effect was observed when *YPT7* knockout was combined with *KAR2* and *LHS1* co-overexpression. Fab titer and yield were increased by up to fourfold, while for IgG, there was no additional improvement.

**Fig. 6:**
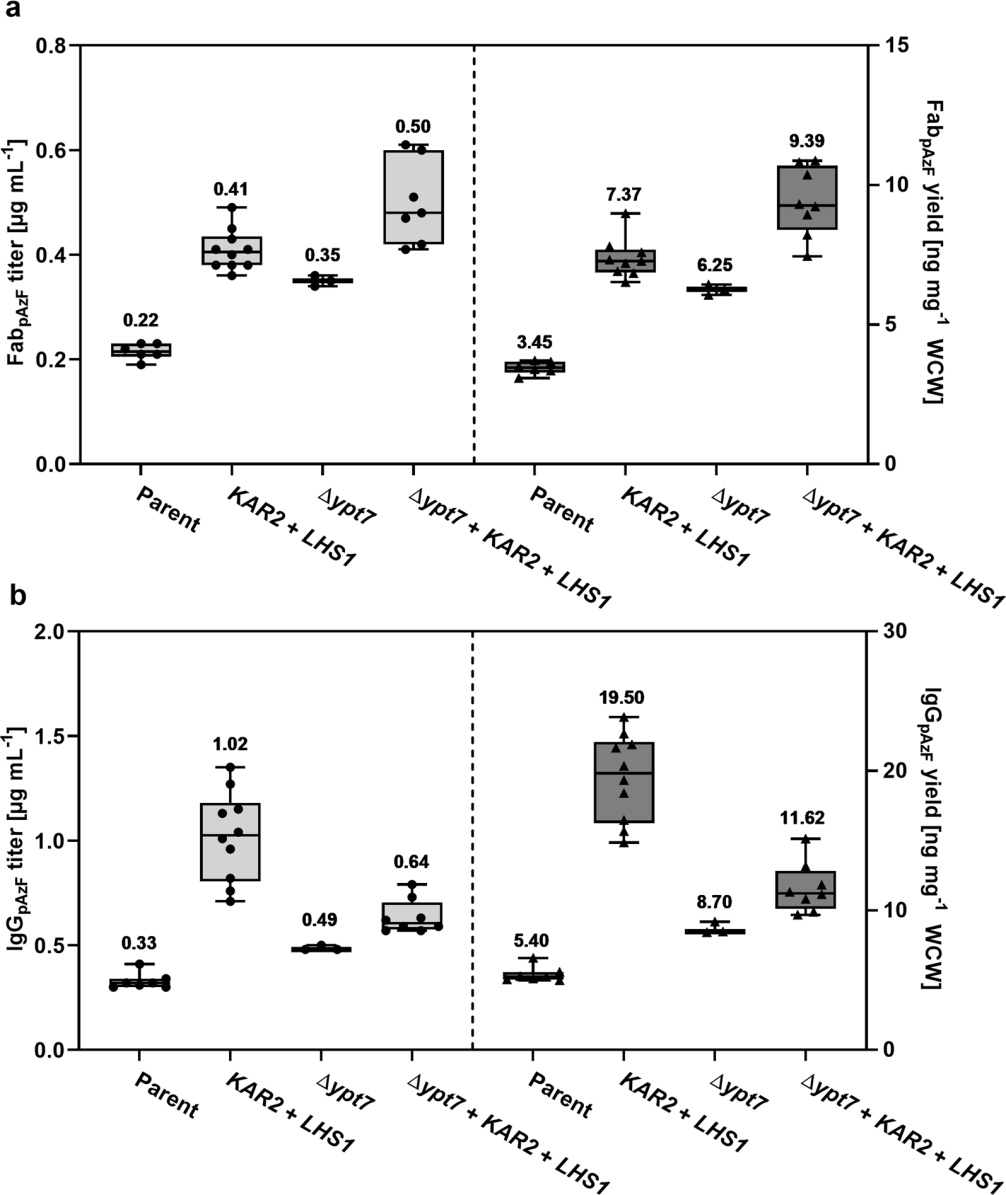
Fab_pAzF_ and IgG_pAzF_ productivity in strains co-overexpressing *KAR2* and *LHS1* with *YPT7* disruption. Titers (left) and yields (right) of **a** Fab_pAzF_ and **b** IgG_pAzF_. Mean values above the boxes were calculated from three to ten biological replicates and are shown as horizontal lines. Dots and triangles show each data point and whiskers show minimal and maximal values. The yields were calculated as the amount of protein per wet cell weight (WCW)

*YPT7* deletion and co-overexpression of *KAR2* and *LHS1* had an additive effect on Fab_pAzF_ production, resulting in a 2.3-fold and a 2.7-fold increase in titer and yield, respectively (Fig. 6a). In the case of IgG_pAzF_, *KAR2* and *LHS1* co-overexpression alone increased protein titer up to threefold and yield up to fourfold (Fig. 6b). In the Δ*ypt7* strain, the co-overexpression increased protein titer up to twofold and yield up to 2.5-fold. For this reason, wild-type strains co-overexpressing *KAR2* and *LHS1* were used for IgG and IgG_pAzF_ production in bioreactors. In all cases, improving co-translational translocation into the ER made major improvements in product secretion.

### Importance of process control for IgG production in *P. pastoris*

The strains co-overexpressing *KAR2* and *LHS1* were used for fed-batch bioreactor cultivations to produce full-length trastuzumab IgG and IgG_pAzF_. Due to the low productivity, three bioreactors were employed for IgG_pAzF_ production and one for IgG production in the parallel DASGIP® system. The cultivation process was adapted from the one previously published by Zavec et al. [11] and the feeding scheme is shown in Additional file 1: Fig. S3. Briefly, selected strains were grown in BSMG until total glycerol consumption (around 22 h) to generate biomass. The feeding strategy involved an 8-h linear addition of 50% glucose, followed by an 18-h linear addition of methanol with a linear decrease in glucose feed rate to adapt the cells to methanol, and continued with a 72-h methanol-only phase for protein production at low growth conditions. Supernatants from four sampling points were analyzed for IgG content and productivity (Additional file 1: Figs. S4 and S5). At the end of the cultivations, supernatants were harvested and used for protein purifications.

**Fig. 7:**
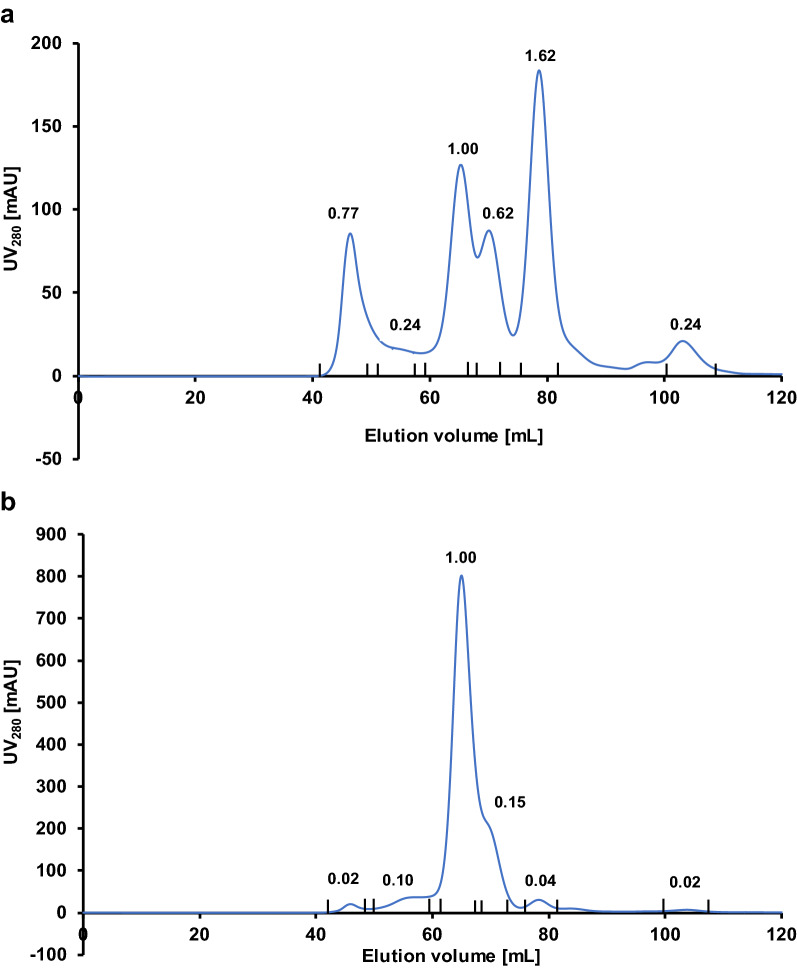
Size-exclusion chromatography elution profiles after protein A affinity purification. **a** IgG_pAzF_ and **b** IgG sample. Integrated peaks were analyzed by SDS-PAGE with silver staining and western blot and pooled. Numbers above the peaks are areas below the curve relative to the area of the peak corresponding to full-length IgG

**Fig. 8:**
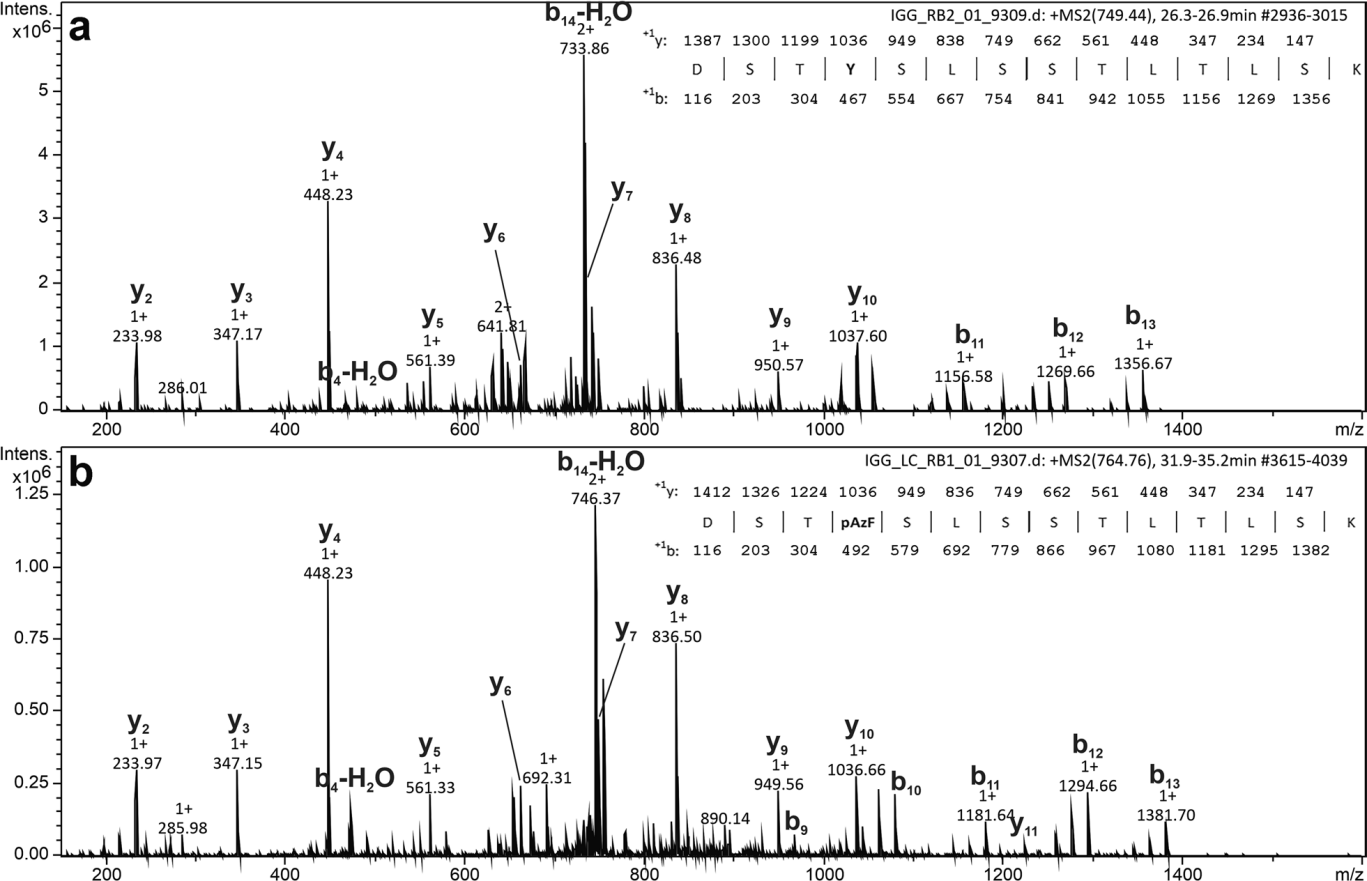
Tandem mass spectrum of the light chain tryptic peptide carrying the site-specific mutation. **a** IgG light chain tryptic peptide with tyrosine at position 173 and **b** IgG_pAzF_ light chain tryptic peptide with pAzF at position 173 (mass increment of 25 Da). All ^+1^y and ^+1^b ions are listed above the corresponding sequence in the upper right corner

Protein A affinity chromatography was used for the first purification step to separate IgG fragments from yeast proteins. Fully assembled IgGs were isolated by size-exclusion chromatography in the next step (Fig. 7). From the SEC profiles, it was possible to recalculate actual tetrameric protein amounts, resulting in around 15 mg L^−1^ for IgG_pAzF_ and 238 mg L^−1^ for IgG. Collected fractions were further analyzed by SDS-PAGE with silver staining and western blotting (Additional file 1: Fig. S6) and those containing pure tetrameric IgGs were used for mass spectrometry analysis to confirm site-specific pAzF incorporation (Fig. 8). However, from intact mass analysis of reduced samples (Additional file 1: Fig. S7), an additional signal for the light chain insensitive to PNGase treatment was observed with an extra 1027 Da corresponding to a propeptide fragment (EEGVSLEKR).

## Discussion

Monoclonal antibody production in microbial hosts is rising in popularity with a growing demand for affordable therapeutics, particularly for treatments of various cancers. *P. pastoris* has already proven to be a promising host cell for recombinant protein production due to its high secretion capacity with low amounts of secreted endogenous proteins, strong inducible promoters, and inexpensive cultivation media. In this work, a *P. pastoris* strain was engineered to improve the production of trastuzumab IgG carrying site-specifically incorporated pAzF as a model uAA. The aim was to further contribute to the development of the yeast as a cost-effective production platform of monoclonal antibodies with an expanded genetic code for the subsequent generation of antibody-drug conjugates.

Since *P. pastoris* has genes for methanol utilization expressed under tightly controllable methanol-inducible promoters, the choice of promoters for expression of all heterologous genes in this work was based on methanol metabolism [15]. The gene for the tRNA_CUA_ was derived from *E. coli* tyrosyl-tRNA, as described previously [21]. It was also established that adding an RNA polymerase II (Pol II) promoter in front of the tRNA_CUA_ gene inserted within the yeast suppressor tRNA (*SUP4*) flanking region with a binding site for RNA polymerase III increased its expression levels. Furthermore, triple tandem copies of the flanked tRNA_CUA_ yielded the highest amber suppression [21]. Therefore, Pol II promoters were tested for the tRNA_CUA_ construct in this work. The modified RS^pAzF^ was derived from *E. coli* tyrosyl-RS [33]. Methanol-inducible promoters for each gene of the orthogonal tRNA_CUA_/RS^pAzF^ pair were used in different combinations to screen for the highest pAzF incorporation into eGFP_Y40X_ as a reporter protein. It was observed that a stronger promoter is needed for tRNA_CUA_ expression to improve pAzF incorporation yield when reverse promoter combinations were compared. Further improvements may be achieved by optimizing the tRNA_CUA_ flanking region and using synthetic promoters for both genes.

Expression levels of both chains constituting tetrameric IgG were tuned using its Fab fragment, which expression and detection are well established in *P. pastoris*. A stronger methanol-inducible promoter for the light chain was more favorable for Fab expression when reverse promoter combinations for each chain were compared. The combinations with strong promoters for both chains resulted in the highest Fab expression. The expression could be further optimized by varying light-to-heavy chain gene copies or screening for synthetic promoters. However, it should be taken into consideration that gene loss can occur due to homologous recombination during long cultivation times if the gene copy number is increased. For this reason, two different promoters and terminators were chosen for the expression of Fab and IgG chains.

The long practice of using the *S. cerevisiae* MFα secretion signal for recombinant protein secretion in *P. pastoris* was challenged. From work by Barrero et al. [24], where a hybrid secretion signal of the oligosaccharide transferase 1 prepeptide and the MFα propeptide was used to improve recombinant protein secretion in *P. pastoris*, it seemed promising to use a different secretion signal for Fab and IgG. Interestingly, the effect of the pre-Ost1-pro-MFα compared to the pre-pro-MFα secretion signal on Fab and IgG secretion was significantly different. The hybrid signal reduced Fab secretion almost twofold and increased IgG secretion 1.3-fold. In order to conclude if this is the general case, more IgGs and their respective Fab fragments should be compared. Nevertheless, in both cases, a significant amount of endogenous proteins was found in culture supernatants when proteins were fused to the pre-pro-MFα secretion signal compared to the pre-Ost1-pro-MFα. Additionally, it would be of great interest to see if the pre-Ost1-pro-MFα can improve the secretion of other glycoproteins compared to the pre-pro-MFα and if it can serve as a starting point for secretion signal sequence engineering.

As a next step in improving recombinant protein secretion, strain engineering was performed by overexpressing genes involved in protein folding and oxidative stress regulation that have shown positive effects in *S. cerevisiae* and *P. pastoris*. Since the effect of overexpression of these factors on Fab production has been studied previously and tetrameric IgG cannot be easily assembled on the screening scale, IgG was used as a model protein. In addition to improving the titer and yield of any IgG format detectable by ELISA, the aim of strain engineering was to improve tetrameric IgG assembly. As only reduced IgG fragments were observed in supernatants from the screenings, the first bottleneck to tackle was the folding of each chain and disulfide bridge formation. Surprisingly, overexpression of foldase Cpr5p, which showed an improvement of IgG production in *S. cerevisiae* [9], and protein disulfide isomerase Pdi1p had adverse effects on IgG secretion, reducing protein titer and yield by 40%. Pdi1p is one of the most abundant proteins in the ER that catalyzes disulfide bond formation and isomerization of incorrectly formed disulfide bonds [34, 35]. A similar effect was observed for IgG production in *S. cerevisiae* [9] and scFv production in *P. pastoris*, where an increase of Kar2p levels upon Pdi1p overproduction was reported [36]. Moreover, it was shown that most unsecreted Fab was degraded in the cytosol before reaching the ER, making ER-associated degradation (ERAD) an explanation of the reduced IgG titers in *PDI1* overexpressing strain less likely [31]. However, no proteasome activity was assayed in this work to test if the reduced titers were due to ERAD.

Overexpression of Hac1p and Yap1p did not improve IgG secretion. Hac1p is a transcription factor regulating the UPR genes and its overexpression showed improvements in the production of secreted recombinant proteins [8, 37, 38]. *YAP1* is another transcription factor involved in oxidative stress relief [39] for which an improved recombinant protein production by *P. pastoris* was reported [26]. However, these effects significantly vary between target products. Furthermore, overexpression of a component of the ER translocon Sbh1p and the ER chaperone Kar2p had no effect on IgG secretion. Positive effects of Sbh1 overexpression on Fab production by *P. pastoris* [28] and α-amylase by *S. cerevisiae* [40] were reported. Since co-overexpression of all translocon subunits on full-length IgG production has not been investigated yet, more detailed experiments are needed to get a clear picture of IgG import into the ER and the role of the translocon components [41]. While overexpression of Lhs1p, a nucleotide exchange factor of Kar2p, which enables ADP to ATP exchange and binding of Kar2p to the next substrate, reduced IgG production by around 60%, co-overexpression of Kar2p and Lhs1p improved IgG production by around 60%. The observed effect is in agreement with previous findings that a major bottleneck in Fab, and therefore, IgG, secretion is the ER import [31].

In addition to overexpression of the common helper factors, strains with disrupted vacuole sorting were prepared by knocking out *YPT7* [28]. In these strains, Fab and IgG production was increased by around twofold. Combining the knockout and co-overexpression of *KAR2* and *LHS1* further increased Fab production by around 3.7-fold, while it resulted in only an additional 10% increase in IgG production compared to the knockout alone. In the case of pAzF-carrying variants, Fab_pAzF_ production showed the same trend as Fab. On the other hand, the best IgG_pAzF_ production was achieved in the strain co-overexpression *KAR2* and *LHS1*, with up to threefold higher productivity than in the parent strain. Productivity of IgG and IgG_pAzF_ formats detectable by ELISA in bioreactor cultivations was around 50- and 20-fold higher than in shake-flask cultivations, respectively. Using a *KAR2* and *LHS1* co-overexpressing strain for IgG and IgG_pAzF_ production in fed-batch bioreactor cultivations yielded significant amounts of tetrameric proteins, up to 238 mg L^−1^ and 15 mg L^−1^, respectively, as calculated from the SEC profiles in the second purification step. Unlike in the screenings, the most abundant species secreted by the strain producing IgG was the tetrameric protein. The strain producing IgG_pAzF_ secreted predominantly free heavy chain since the light chain translation was reduced due to the internal amber codon. The ratio of produced tetrameric IgG_pAzF_ to the free heavy chain could be increased by downregulation or disruption of nonsense-mediated mRNA decay [42]. Trials to downregulate or replace the eukaryotic peptide release factor 1 with variants that preferentially recognize opal and ochre stop codons did not improve pAzF incorporation (not shown).

Upon mass spectrometry analysis of purified IgG and IgG_pAzF_ fractions, intact mass measurements showed an additional light chain signal with an extra 1027 Da. Since the signal was present in samples treated and untreated with PNGase, we concluded that it belonged to the propeptide fragment (EEGVSLEKR), which was cleaved off upon tryptic digest and was not detected by peptide mass fingerprinting. This leaves some room for further investigation if the observed leftover is due to cell lysis, causing some IgG to have the partially processed light chain in the supernatant, or a mixture of IgG with fully and partially processed light chain being secreted throughout the cultivation. Additionally, process optimization by employing methanol feed dependent on the levels of dissolved oxygen [43] could further reduce cellular stress which frequently causes incomplete secretion signal processing.

## Conclusions

In this work, a new contribution to strain engineering of *P. pastoris* for the production of monoclonal antibodies with the expanded genetic code was made. By using a promising fusion secretion signal, pre-Ost1-pro-MFα [24, 25], co-overexpressing *KAR2* and *LHS1,* and employing controlled cultivation conditions, full-length trastuzumab IgG with pAzF in the light chain was produced in titers up to 15 mg L^−1^. Production of trastuzumab Fab with pAzF in the light chain was further improved by deleting *YPT7*, causing the disruption of vacuolar targeting and increasing product titer and yield almost fourfold. Additional improvements could potentially be achieved by disrupting nonsense-mediated mRNA decay and, in the case of IgG, glycoengineering of *P. pastoris* to obtain a more human-like glycosylation pattern. Furthermore, to reduce the observed propeptide leftover in part of the secreted product, an alternative methanol-feed strategy should be employed, such as pulse-feed controlled by the levels of dissolved oxygen. Lastly, in this work, the major bottleneck of IgG production was found to be the ER import, which is in agreement with previous research on Fab production in *P. pastoris* [31].

## Materials and methods

### Preparation of *P. pastoris* strains

All plasmids were prepared in a series of GoldenGate assemblies as described previously [23] and cloning of tRNA_CUA_/RS^pAzF^ and Fab/IgG expression plasmids is shown in Additional file 1: Fig. S1. Primers used for cloning heterologous genes, amplification of overexpression targets and gene deletions are listed in Additional file 2: Table S2. All the *P. pastoris* strains used in this study were derived from the wild-type CBS2612 strain [44] and are listed with their respective antibiotic resistances in Additional file 2: Table S3. The strain used as a positive control in eGFP screenings expressed the protein under the P_*AOX1*_ (P_*AOX1*__eGFP), the same as the strain expressing eGFP with the internal amber stop codon at the position Y40 (P_*AOX1*__eGFP_Y40X_). The P_*AOX1*__eGFP_Y40X_ strain also expressed the orthogonal pair of the suppressor tRNA_CUA_ and engineered RS^pAzF^ [3], for which combinations of methanol-inducible promoters were tested in this work (P_*X*__*RS*^pAzF^_P_*Y*__3x*SUP4*-tRNA_CUA_). The tRNA_CUA_ expression cassette contained three tandem copies of the tRNA lacking the 3′-CCA terminus and each copy was flanked by the *SUP4* region from *S. cerevisiae* [21]. The strain used for Fab screenings expressed both chains under different methanol-inducible promoters (P_*X′_LC_PY′*__*HC*-Fab). Each chain was fused to the pre-Ost1-pro-MFα secretion signal [24, 25] lacking the C-terminal EAEA sequence. The final producer strain expressed IgG fused to the same secretion signal. The light chain was expressed from the P_*AOX1*_ and the heavy chain from the P_*DAS2*_ (P_*AOX1*__*LC*_P_*DAS2*__*HC*-IgG). The strain expressing Fab and IgG variant with the amber codon in the light chain at position Y173 (P_*AOX1*__*LC*_Y173X__P_*DAS2*__*HC*-Fab; P_*AOX1*__*LC*_Y173X__P_*DAS2*__*HC*-IgG) also expressed tRNA_CUA_ under the P_*DAS2*_ and the synthetase under the P_*AOX1*_ (P_*AOX1*__*RS*^pAzF^_P_*DAS2*__3x*SUP4*-tRNA_CUA_). Helper factors *PDI1* (PP7435_Chr4-0107), *HAC1*i (PP7435_Chr1-0700), *YAP1* (PP7435_Chr4-0370), and *KAR2* (PP7435_Chr2-1167) were constitutively overexpressed under the glyceraldehyde-3-phosphate dehydrogenase promoter (P_*GAP*_). *LHS1* (PP7435_Chr1-0059) was overexpressed under the promoter for mitochondrial porin 1 (P_*POR1*__*LHS1*) and *CPR5* (PP7435_Chr1-0581) and *SBH1* (PP7435_Chr2-0220) under the promoter for the translational elongation factor EF-1α (P_*TEF*__*CPR5*; P_*TEF*__*SBH1*). The *YPT7* (PP7435_Chr4-0052) deletion strains were prepared from parents producing both variants of Fab and IgG and a positive clone from each was selected for *KAR2* and *LHS1* co-overexpression.

### Screenings in 24 deep-well plates

All the screenings were performed in 24 deep-well plates with shaking at 280 rpm at 25 °C. The minimal medium used for main cultures was ASMv6, as previously described [11] and controlled glucose release was achieved using the EnPump200 kit (EnPresso) to the final polysaccharide concentration of 25 g L^−1^ and 0.3% of the corresponding enzyme. For the precultures, 2 mL of selective YPD media (10 g L^−1^ yeast extract, 20 g L^−1^ soy peptone, 2% v/v glucose) were inoculated with single colonies from the selective YPD agar (YPD medium with 2% agar) plates after incubation for 2 days at 30 °C. After 28 h, the precultures were washed and 2 mL of the main cultures were inoculated to a starting OD_600_ of 8. All the cultures expressing heterologous proteins with pAzF were supplemented with 2 mM pAzF dissolved in 2 mM NaOH and the negative controls with 2 mM NaOH. Methanol shots were given to the cells for induction: 0.5% was given 3 h post-inoculation and three shots of 1% 19, 27, and 43 h post-inoculation. The cultures were harvested 48 h post-inoculation by taking 1 mL cultures and centrifuging them at 13 000 rpm for 5 min. Wet cell weight was measured from cell pellets and the supernatant was analyzed by ELISA (Fab and IgG cultures). For eGFP expression measurements, cultures from the end of the screenings were diluted in pre-cooled PBS (0.24 g L^−1^ KH_2_PO_4_, 1.8 g L^−1^ Na_2_HPO_4_∙2H_2_O, 0.2 g L^−1^ KCl, 8 g L^−1^ NaCl, pH 7.4) to an OD_600_ of 0.4 in 96 microtiter plates.

### Flow cytometry analysis

Flow cytometry analysis was performed in a CytoFLEX S benchtop flow cytometer (Beckman Coulter). The excitation wavelength for eGFP was 488 nm and for detection, a 525/40 nm bandpass filter was used. The sample flow rate was set to 30 µL min^−1^, and the number of recorded events was 10,000. For statistical analysis, geometric means of the fluorescence and the forward scatter signal heights were taken from which a mean relative cell-associated product (*P*_Rel_) was calculated as reported previously [45, 46]. The percentage of stop codon suppression in eGFP_Y40X_ was calculated from the ratio of the *P*_Rel_ of each clone and *P*_Rel_ of two to three biological replicates of the positive control.

### Enzyme-linked immunosorbent assay

ELISA was performed as described previously [31], except for the coating antibodies used. For Fab samples, mouse anti-human IgG Fab (Invitrogen) was used and for IgG samples, anti-human IgG (γ-chain specific, Sigma) was used. The standard for Fab was as described, while the standard for IgG was the commercial Herceptin™ (Roche). Stop codon suppression yields were calculated from the product yields with pAzF for each clone relative to two to three biological replicates expressing corresponding wild-type proteins. Titer and yield fold changes in overexpression screenings were calculated from the ratios of titers and yields for each clone relative to the average of titers and yields of two to three biological replicates of the wild-type strains.

### Silver staining and western blot

For SDS-PAGE, either NuPAGE 12% Bis-Tris (for Fab samples) or NuPAGE® Novex 4-12% Bis-Tris (for IgG samples) gels were used. Samples were prepared by boiling 13 or 15 µL supernatants with 5 µL loading buffer (4×, NuPAGE) with or without 2 µL reducing agent (10×, NuPAGE) or 5 mM *N*-ethylmaleimide (NEM, Thermofisher), respectively, at 100 °C for 1 min. Samples were run in 1×MOPS (NuPAGE) at 180 V for 1 h. After SDS-PAGE run, gels were fixed in a fixing solution (10% v/v acetic acid, 50% v/v ethanol) with gentle agitation overnight at 4 °C. The next day, the fixing solution was discarded, and gels were incubated in an incubation solution (68.2 g L^−1^ sodium acetate, 3.2 g L^−1^ Na_2_S_2_O_3_·5H_2_O, 30% v/v ethanol, 0.25% v/v glutaraldehyde) with gentle agitation for 30 min at room temperature. Gels were then washed three times with distilled water for 10 min. Silver nitrate solution (0.1% w/v AgNO_3_, 0.02% v/v formaldehyde) was added and gels were incubated for 20 min at room temperature. After the binding, gels were briefly washed with distilled water and a developing solution (5.0 g L^−1^ Na_2_CO_3_, 0.01% v/v formaldehyde) was added. Gels were incubated at room temperature until protein bands were of desired intensity. Further development was quenched with 50 mM EDTA and gels were scanned. Gels that were not used for silver staining were blotted on Trans-Blot turbo mini 0.2 µm nitrocellulose membranes (Bio-Rad) in the Trans-Blot Turbo transfer system (Bio-Rad). Membranes were immediately incubated in blocking buffer (PBS pH 7.4, 0.05% Tween, 2% BSA) at 4 °C with gentle agitation overnight. The next day, membranes were washed three times for 5 min with washing buffer (PBS pH 7.4, 0.05% Tween). Primary antibodies diluted in the blocking buffer were incubated for 1 h at room temperature with a washing step in between. Secondary fluorescent antibodies were incubated in the same way, and after the final washing step, fluorescence was detected by the Chemidoc MP imaging system (Bio-Rad).

### Fed-batch bioreactor cultivation

The bioreactor cultivations were done in a DASGIP® Parallel Bioreactor System (Eppendorf AG) in 1 L bioreactor vessels. Process parameters were controlled remotely by the DASGIP control software (Eppendorf AG, Germany). During the batch phase, pH was maintained at 5.5, while during the feed phases, at 5.0 by 25% NH_4_OH. The temperature was held at 25 °C during the whole process. Dissolved oxygen (DO) was controlled by a DO-cascade, which consisted of a sequential increase of stirring speed from 200 to 1250 rpm, ingas flow rate from 6 to 50 sL h^−1^ and ingas oxygen concentration between 21 and 100%, depending on individual strain demand. Precultures were inoculated from 1 mL working cell banks in 50 mL selective YPG in 300 mL shake flasks and cultivated at 25 °C overnight with mixing at 180 rpm. Before the bioreactor inoculation, OD_600_ was measured and the appropriate volume of the precultures was washed with the batch medium. The batch volume of 300 mL was inoculated to an OD_600_ of 1 and 2 mM pAzF were added. The batch phase of the bioreactor cultivations took approximately 24 h. The second phase of linear feed with 50% glucose (rate of feed calculated as y = 0.225x + 1.95, x: time [h]) was initiated when the total glycerol was consumed. After 8 h, the third phase was initiated by the linear increase in 100% methanol (y = 0.028x + 0.6) and the simultaneous linear decrease in 50% glucose (y = 3.75 − 0.111x). After 18 h of the co-feed phase, the fourth phase was initiated by the linear feed with 100% methanol alone (y = 0.028x + 1.1) [11]. During the bioreactor cultivations, samples were taken at six time points. Two milliliters were taken in three technical replicates for each bioreactor and centrifuged at 4 °C with a speed of 13,000 rpm for 5 min. Pellets were washed with 1 mL 0.1 M HCl and dried in preweighted tubes at 105 °C for determination of yeast dry mass (YDM). Supernatants were used for the determination of protein concentration. The cultivations were terminated 90 h post-induction by harvesting supernatants of each bioreactor at 4 °C with a speed of 10000×*g* for 30 min. The harvested supernatants were used for protein purification.

### Protein purification

Bioreactor supernatants were concentrated by tangential flow filtration and the buffer was changed to PBS (pH 7.4). Affinity purification was performed using the HiTrap Protein A HP column. The column was equilibrated with 12 column volumes (CVs) binding buffer (PBS, pH 7.4) at the rate of 1 mL min^−1^. The sample was loaded at the rate of 0.5 mL min^−1^ and washed with the binding buffer at 1 mL min^−1^ for 15 CVs. Elution steps were done at the rate of 1 mL min^−1^ using 0.1 M glycin-HCl (pH 3.5) as the elution buffer, starting with a 10 CVs linear gradient from 0 to 50% elution buffer, followed by 5 CVs with 50% elution buffer. A linear gradient from 50 to 100% elution buffer was applied for 10 CVs or until the absorbance at 280 nm started rising. Elution at 100% elution buffer with pH 2.5 was applied for the last 12 CVs. Each collected fraction was neutralized with 100 mM Tris-HCl. Fractions that showed increased absorbance, washed fractions and unbound fractions were analyzed by SDS-PAGE. Those containing full-length IgG were pooled and buffer was exchanged to PBS (pH 7.4) using Amicon spin filters. The second step of size-exclusion purification was performed using HiLoad Superdex 16/600 column. Equilibration and washing buffer was PBS (pH 7.4). The flow rate applied was 0.5 mL min^−1^. Fractions belonging to the same peak were pooled and analyzed by SDS-PAGE and western blot.

### Mass spectrometry analysis

Fractions containing pure full-length IgGs were analyzed by mass spectrometry. The proteins were reduced with 2.5 mM DTT and incubated at 37 °C for 30 min. Additionally, an aliquot of each sample was de-*N*-glycosylated by the addition of 2.5 mU PNGase F and incubated at 40 °C for 2 h.

Two µg of the protein was directly injected into an LC-ESI-MS system (LC: Agilent 1290 Infinity II UPLC). A gradient from 15 to 80% acetonitrile in 0.1% formic acid [using a Waters BioResolve column (2.1 × 5 mm)] at a flow rate of 400 µL min^−1^ was applied (15-min gradient time). Detection was performed with a Q-TOF instrument (Agilent Series 6560 LC-IMS-QTOFMS) equipped with the Jetstream ESI source in positive ion, MS mode (range: 100–3200 Da). Instrument calibration was performed using an ESI calibration mixture (Agilent). Data were processed using MassHunter BioConfirm B.08.00 (Agilent) and the spectrum was deconvoluted by MaxEnt.

Alternatively, the samples were digested with trypsin (Promega, Trypsin Gold) after S-alkylation with iodoacetamide. The digested samples were loaded on a nanoEase C18 column (nanoEase M/Z HSS T3 Column, 100 Å, 1.8 µm, 300 µm × 150 mm, Waters) using 0.1% formic acid as the aqueous solvent. A gradient from 3.5% B (B: 80% ACN, 20% A) to 40% B in 30 min was applied, followed by a 5-min gradient from 40% B to 95% B that facilitated the elution of large peptides at a flow rate of 6 µL min^−1^. Detection was performed with an ion-trap MS (amazon speed ETD, Bruker) equipped with the standard ESI source in positive ion, DDA mode (= switching to MSMS mode for eluting peaks). MS-scans were recorded (range: 150–2200 Da) and the 8 highest peaks were selected for fragmentation. Instrument calibration was performed using an ESI calibration mixture (Agilent).

The analysis files were converted (using Data Analysis, Bruker) to mgf files, which are suitable for performing an MS/MS ion search with X!-Tandem. The files were searched against a *P. pastoris* database including the following sequences, adding 25.00648 Da at Y as a potential modification to allow the identification of the pAzF incorporation.

## Supplementary Information

**Additional file 1: Supplementary figures. Figure S1.** Cloning scheme of plasmids containing **a** tRNA_CUA_/RS^pAzF^ pair and **b** Fab/IgG heavy and light chains using Golden*Pi*CS. Backbone 1 (BB1) carried either promoter (fusion sites 1 and 2; green arrows), recombinant gene (fusion sites 2 and 3; blue and yellow arrows), or terminator (fusion sites 3 and 4; gray arrows). Three tandem copies of *SUP4*-tRNA_CUA_ were generated in a single BB1 assembly from PCR products carrying custom fusion sites (FS) in overhangs in BsaI/T4 mediated restriction/ligation as shown above BB1_3x *SUP4*-tRNA_CUA_. In the case of Fab/IgG with the amber stop codon at the position Y173 in the light chain, a custom fusion site (black and grey boxes) was created to replace 5′ TAC of Y173 to 5′ TAG as shown in the insert above BB1_*LC*. Overhangs of the PCR product carried recognition sites for BsaI (white boxes) for BB1 assembly. BB1s were assembled into BB2s containing fusion sites 1 and 4 flanked by the fusion sites A and B or B and C in BbsI/T4 mediated restriction/ligation. BB2s carried single expression cassettes and were assembled into BB3s with fusion sites A and C to accommodate two expression cassettes. Details about the fusion site choice were elaborated by Kolb et al. [1]. The figure was generated on Biorender.com. **Figure S2.** SDS-PAGE with silver staining (left) and western blot (right) analysis of the supernatants from the screenings: **a** Fab and **b** IgG producing strains. Two biological replicates of each strain were used for the analyses and one of each was used for the reduced protein analysis (DTT “+” wells). The protein ladder was loaded in the first well, and the corresponding protein standards were loaded in the second well. The pre-pro-MFα secretion signal is abbreviated as “ppM” and the pre-Ost1-pro-MFα as “pOpM”. The green bands show the heavy chain and the red ones the light chain. Due to the high standard concentration, in-gel fragmentation is observed. **Figure S3.** Feeding strategy for methanol-inducible fed-batch bioreactor cultivations. A defined minimal medium containing 2% glycerol is used in the batch phase, where cells generate biomass before methanol induction. Upon total glycerol consumption, cells are slowly fed with 50% glucose (blue line) for eight hours, after which the glucose feed slows down and 100% methanol (red line) is slowly added to pre-condition the cells. This co-feed phase takes 18 h. The last cultivation phase takes 3 days and only 100% methanol is slowly fed to the cells. **Figure S4.** IgG and IgG_pAzF_ titers (bars) and yields (lines) produced by the *KAR2* and *LHS1* co-overexpressing strains during the methanol induction phase of fed-batch bioreactor cultivations. The yields were calculated as the amount of protein per yeast dry mass (YDM). Mean values above the bars were calculated from three technical replicates for IgG samples and one technical replicate from three bioreactors for the IgG_pAzF_ samples. Error bars represent standard deviations and are not shown for the yields to avoid graph crowdedness. **Figure S5.** **a** SDS-PAGE with silver staining and **b** western blot of supernatants from the fed-batch bioreactor cultivations of the IgG and IgG_pAzF_ producing strains. The cultivation medium for IgG production did not contain pAzF (marked with minuses), while the media for IgG_pAzF_ production contained 1 mM pAzF (marked with pluses). The first and the last lane contain the protein ladder. The lane with the commercial trastuzumab standard is labeled with “St” and the numbers are the sample numbers. The red bands show the light chain (LC), while the green bands show the heavy chain (HC). The main fragmentation products are assigned on the right. **Figure S6.** Analysis of selected fractions after IgG_pAzF_ and IgG purification by size-exclusion chromatography. **a** SDS-PAGE with silver staining and **b** western blot. One microgram of total protein was loaded in each well so the band intensity does not reflect the relative protein amounts in the samples. The first and the middle lane contain the protein ladder. The lane with a commercial trastuzumab standard is labeled with “St”. The red bands show the light chain, while the green bands show the heavy chain. **Figure S7.** Deconvoluted MS spectra of the reduced IgG_pAzF_ **a**, **b** and IgG **c**, **d** and treated with PNGase **b**, **d**. The molecular weight of the light chain without pAzF is 23443.1 Da and 23468.1 Da of the pAzF-carrying variant (in the gray box). The molecular weight of the unglycosylated heavy chain is 49284.65 Da (in the golden box).

**Additional file 2: Supplementary tables. Table S1.** Concentration test for optimal amber suppression in eGFP_Y40X_ by pAzF. Wet cell weight was determined at the end of 24 deep-well plate screenings by weighing the pellets from 1 mL culture aliquots. Negative control cultures contained varying amounts of NaOH while the suppression cultures contained varying amounts of pAzF, prepared as a stock solution of 100 mM in 100 mM NaOH. The mean relative cell-associated product was calculated from the ratio of the geometric mean of fluorescence intensity and forward scatter as described by Kolb et al. [1] and Dumas et al. [2]. Average values and the corresponding standard deviations were calculated from three biological replicates. **Table S2.** List of primers prepared for this study. **Table S3.** List of strains prepared in this study.

## Data Availability

The datasets and materials used and/or analyzed during the current study are available from the corresponding author on reasonable request.
