# Supplementary material for: From strain engineering to process development: monoclonal antibody production with an unnatural amino acid in Pichia pastoris
Source: Microb Cell Fact. 2022 Aug 11;21:157. doi: 10.1186/s12934-022-01882-6 (PMC9367057; doi:10.1186/s12934-022-01882-6)
Supplement: Supplementary file 1 — Additional file 1: Supplementary figures. Figure S1. Cloning scheme of plasmids containing a tRNACUA/RSpAzF pair and b Fab/IgG heavy and light chains using GoldenPiCS. Backbone 1 (BB1) carried either promoter (fusion sites 1 and 2; green arrows), recombinant gene (fusion sites 2 and 3; blue and yellow arrows), or terminator (fusion sites 3 and 4; gray arrows). Three tandem copies of SUP4-tRNACUA were generated in a single BB1 assembly from PCR products carrying custom fusion sites (FS) in overhangs in BsaI/T4 mediated restriction/ligation as shown above BB1_3x SUP4-tRNACUA. In the case of Fab/IgG with the amber stop codon at the position Y173 in the light chain, a custom fusion site (black and grey boxes) was created to replace 5′ TAC of Y173 to 5′ TAG as shown in the insert above BB1_LC. Overhangs of the PCR product carried recognition sites for BsaI (white boxes) for BB1 assembly. BB1s were assembled into BB2s containing fusion sites 1 and 4 flanked by the fusion sites A and B or B and C in BbsI/T4 mediated restriction/ligation. BB2s carried single expression cassettes and were assembled into BB3s with fusion sites A and C to accommodate two expression cassettes. Details about the fusion site choice were elaborated by Kolb et al. [1]. The figure was generated on Biorender.com. Figure S2. SDS-PAGE with silver staining (left) and western blot (right) analysis of the supernatants from the screenings: a Fab and b IgG producing strains. Two biological replicates of each strain were used for the analyses and one of each was used for the reduced protein analysis (DTT “+” wells). The protein ladder was loaded in the first well, and the corresponding protein standards were loaded in the second well. The pre-pro-MFα secretion signal is abbreviated as “ppM” and the pre-Ost1-pro-MFα as “pOpM”. The green bands show the heavy chain and the red ones the light chain. Due to the high standard concentration, in-gel fragmentation is observed. Figure S3. Feeding strategy for methanol [file 12934_2022_1882_MOESM1_ESM.pdf]

From strain engineering to process development: monoclonal antibody production with an unnatural amino acid in *Pichia pastoris*

Nora Tir[1,2], Lina Heistinger[1,2]*, Clemens Grünwald-Gruber[3], Leo A. Jakob[4], Stephan Dickgiesser[5], Nicolas Rasche[5], Diethard Mattanovich[2]**

[1] University of Natural Resources and Life Sciences, Department of Biotechnology, Christian Doppler Laboratory for Innovative Immunotherapeutics, Muthgasse 18, 1190 Vienna, Austria
[2] University of Natural Resources and Life Sciences, Department of Biotechnology, Institute of Microbiology and Microbial Biotechnology, Muthgasse 18, 1190 Vienna, Austria
[3] University of Natural Resources and Life Sciences, Vienna Core Facility Mass Spectrometry Muthgasse 18, 1190 Vienna, Austria
[4] University of Natural Resources and Life Sciences, Department of Biotechnology, Institute of Bioprocess Science and Engineering, Muthgasse 18, 1190 Vienna, Austria
[5] ADCs & Targeted NBE Therapeutics, Merck Healthcare KGaA, Frankfurter Str. 250, 64293 Darmstadt, Germany
* present address: ETH Zürich, Department of Biology, Institute of Biochemistry, 8093 Zürich, Switzerland

**Corresponding author:
Univ. Prof. Dr. Diethard Mattanovich
University of Natural Resources and Life Sciences, Department of Biotechnology, Institute of Microbiology and Microbial Biotechnology
Muthgasse 18, 1190 Vienna, Austria
Email: diethard.mattanovich@boku.ac.at
Phone: +43 1 47654 79006; FAX: +43 1 47654 79009

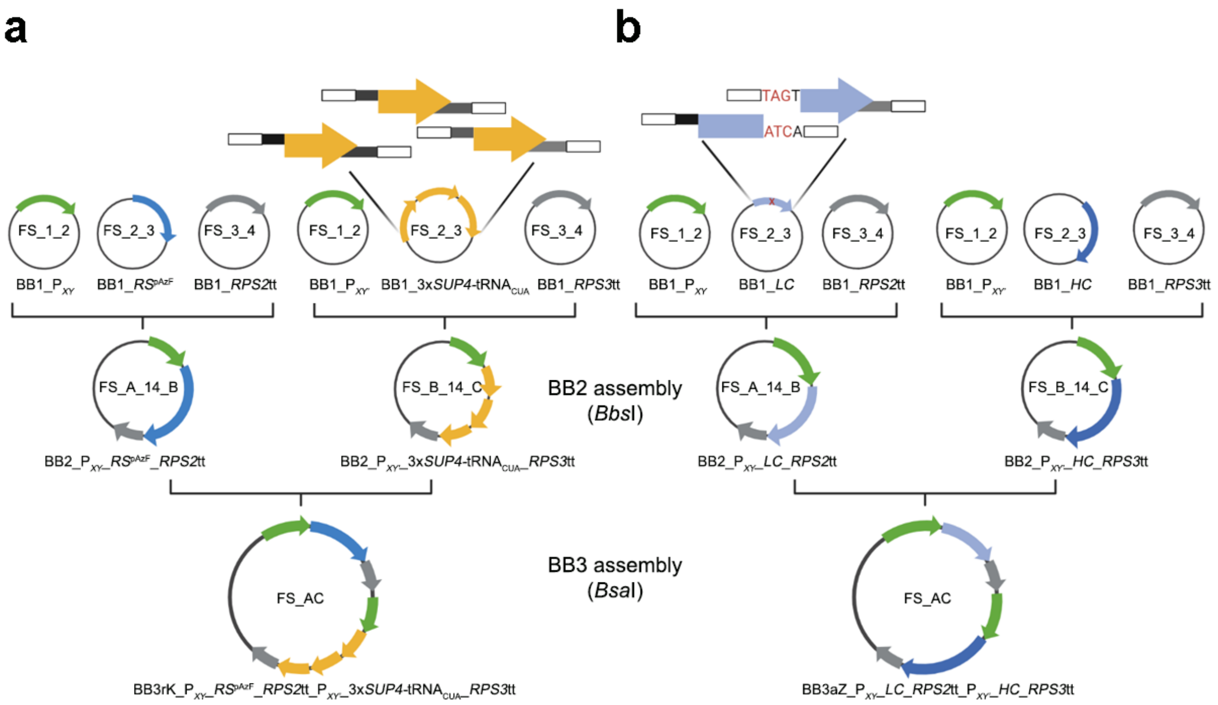

Fig. S1. Cloning scheme of plasmids containing **a** $tRNA_{CUA}/RS^{pAzF}$ pair and **b** Fab/IgG heavy and light chains using Golden*Pi*CS. Backbone 1 (BB1) carried either promoter (fusion sites 1 and 2; green arrows), recombinant gene (fusion sites 2 and 3; blue and yellow arrows), or terminator (fusion sites 3 and 4; gray arrows). Three tandem copies of *SUP4*-$tRNA_{CUA}$ were generated in a single BB1 assembly from PCR products carrying custom fusion sites (FS) in overhangs in *Bsa*I/T4 mediated restriction/ligation as shown above BB1_3x *SUP4*-$tRNA_{CUA}$. In the case of Fab/IgG with the amber stop codon at the position Y173 in the light chain, a custom fusion site (black and grey boxes) was created to replace 5' TAC of Y173 to 5' TAG as shown in the insert above BB1_*LC*. Overhangs of the PCR product carried recognition sites for *Bsa*I (white boxes) for BB1 assembly. BB1s were assembled into BB2s containing fusion sites 1 and 4 flanked by the fusion sites A and B or B and C in *Bbs*I/T4 mediated restriction/ligation. BB2s carried single expression cassettes and were assembled into BB3s with fusion sites A and C to accommodate two expression cassettes. Details about the fusion site choice were elaborated by [1]. The figure was generated on Biorender.com.

References:

1. Prielhofer R, Barrero JJ, Steuer S, Gassler T, Zahrl R, Baumann K, Sauer M, Mattanovich D, Gasser B, Marx H. Golden*Pi*CS: A Golden Gate-Derived Modular Cloning System for Applied Synthetic Biology in the Yeast *Pichia Pastoris*. BMC Syst Biol. 2017;11.

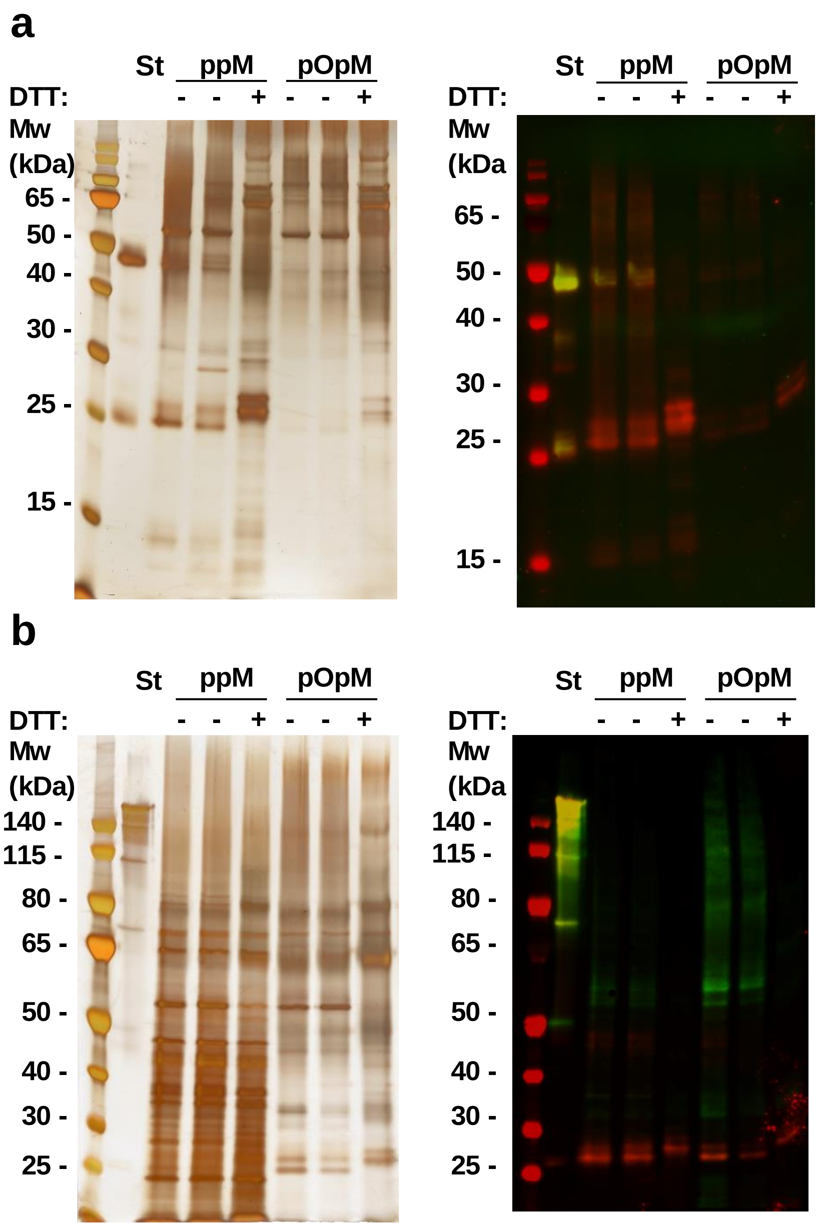

Fig. S2. SDS-PAGE with silver staining (left) and western blot (right) analysis of the supernatants from the screenings: **a** Fab and **b** IgG producing strains. Two biological replicates of each strain were used for the analyses and one of each was used for the reduced protein analysis (DTT "+" wells). The protein ladder was loaded in the first well, and the corresponding protein standards were loaded in the second well. The pre-pro-MFα secretion signal is abbreviated as "ppM" and the pre-Ost1-pro-MFα as "pOpM". The green bands show the heavy chain and the red ones the light chain. Due to the high standard concentration, in-gel fragmentation is observed.

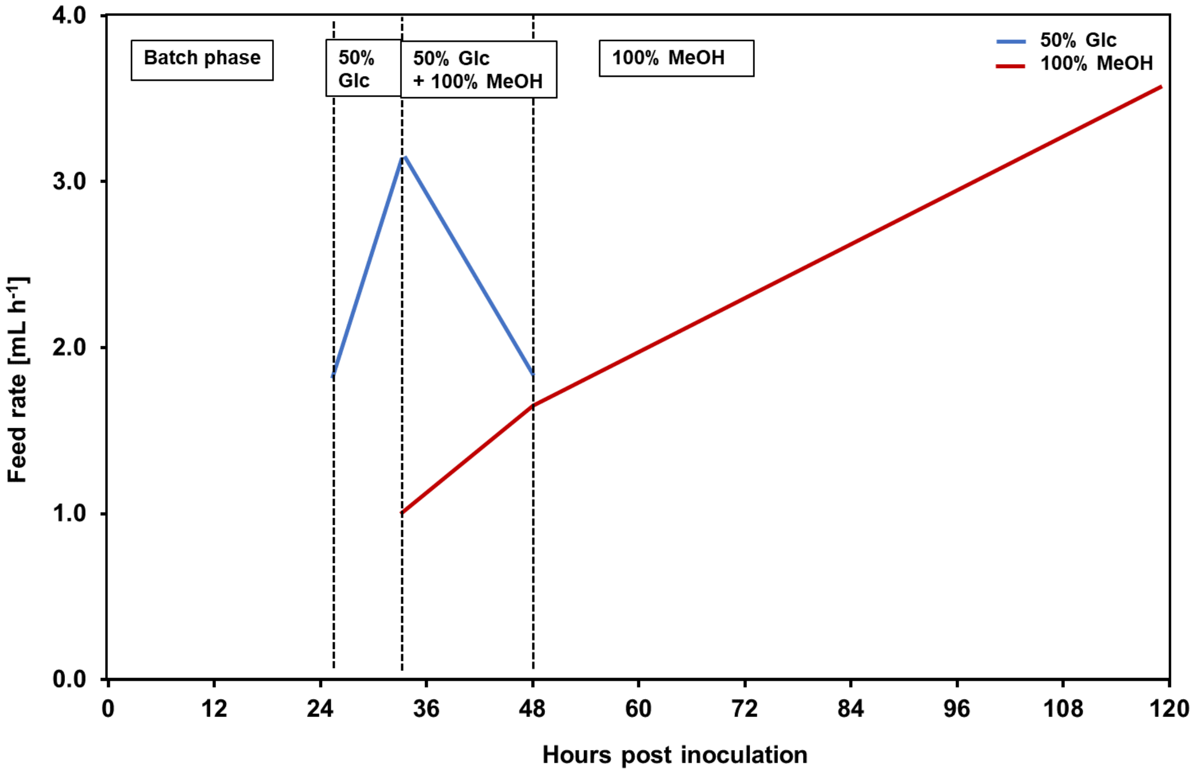

Fig. S3. Feeding strategy for methanol-inducible fed-batch bioreactor cultivations. A defined minimal medium containing 2% glycerol is used in the batch phase, where cells generate biomass before methanol induction. Upon total glycerol consumption, cells are slowly fed with 50% glucose (blue line) for eight hours, after which the glucose feed slows down and 100% methanol (red line) is slowly added to pre-condition the cells. This co-feed phase takes 18 hours. The last cultivation phase takes three days and only 100% methanol is slowly fed to the cells.

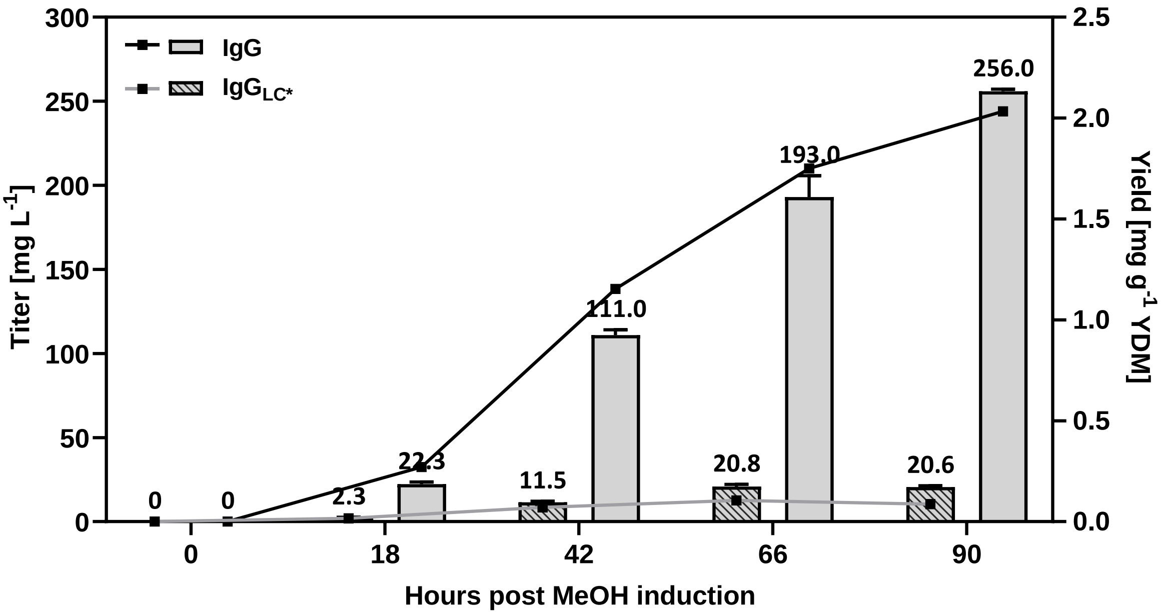

Fig. S4. IgG and IgG_{pAzF} titers (bars) and yields (lines) produced by the *KAR2* and *LHS1* co-overexpressing strains during the methanol induction phase of fed-batch bioreactor cultivations. The yields were calculated as the amount of protein per yeast dry mass (YDM). Mean values above the bars were calculated from three technical replicates for IgG samples and one technical replicate from three bioreactors for the IgG_{pAzF} samples. Error bars represent standard deviations and are not shown for the yields to avoid graph crowdedness.

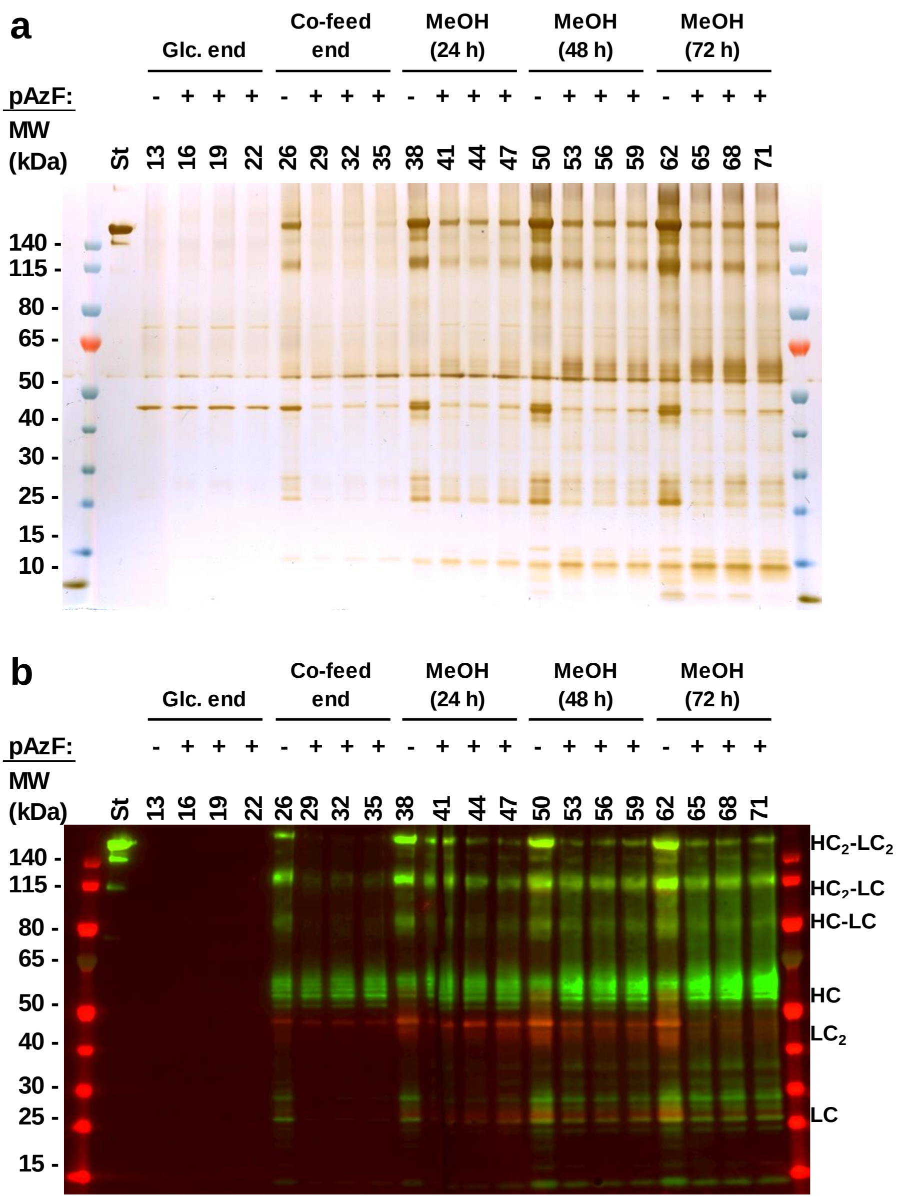

Fig. S5. a SDS-PAGE with silver staining and **b** western blot of supernatants from the fed-batch bioreactor cultivations of the IgG and IgG_{pAzF} producing strains. The cultivation medium for IgG production did not contain pAzF (marked with minuses), while the media for IgG_{pAzF} production contained 1 mM pAzF (marked with pluses). The first and the last lane contain the protein ladder. The lane with the commercial trastuzumab standard is labeled with "St" and the numbers are the sample numbers. The red bands show the light chain (LC), while the green bands show the heavy chain (HC). The main fragmentation products are assigned on the right.

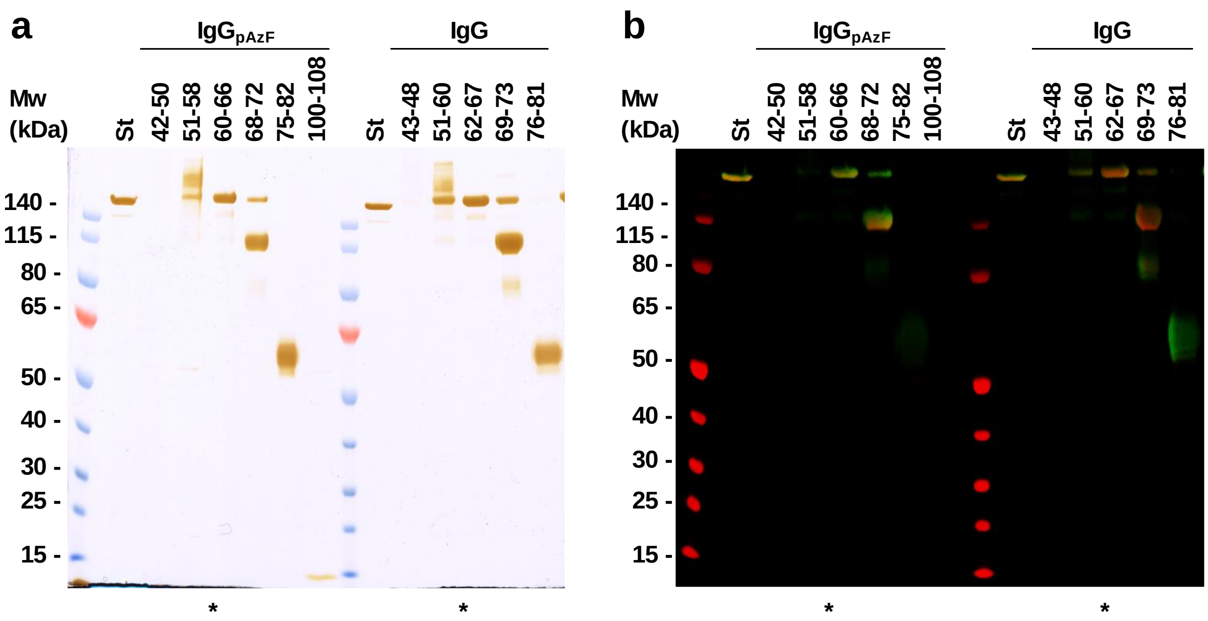

Fig. S6. Analysis of selected fractions after IgG$_{pAzF}$ and IgG purification by size-exclusion chromatography. **a** SDS-PAGE with silver staining and **b** western blot. One microgram of total protein was loaded in each well so the band intensity does not reflect the relative protein amounts in the samples. The first and the middle lane contain the protein ladder. The lane with a commercial trastuzumab standard is labeled with "St". The red bands show the light chain, while the green bands show the heavy chain.

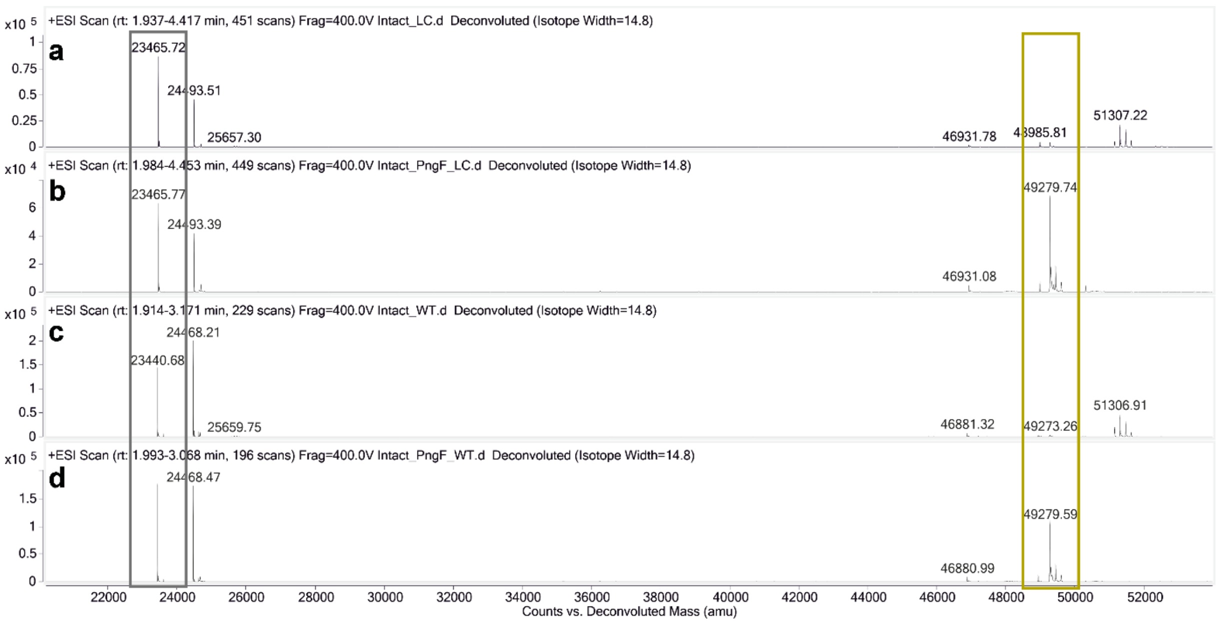

Fig. S7. Deconvoluted MS spectra of the reduced IgG$_{pAzF}$ **a**, **b** and IgG **c**, **d** and treated with PNGase **b**, **d**. The molecular weight of the light chain without pAzF is 23443.1 Da and 23468.1 Da of the pAzF-carrying variant (in the gray box). The molecular weight of the unglycosylated heavy chain is 49284.65 Da (in the golden box).
