# Supplementary material for: From strain engineering to process development: monoclonal antibody production with an unnatural amino acid in Pichia pastoris
Source: Microb Cell Fact. 2022 Aug 11;21:157. doi: 10.1186/s12934-022-01882-6 (PMC9367057; doi:10.1186/s12934-022-01882-6)
Supplement: Supplementary file 2 — Additional file 2: Supplementary tables. Table S1. Concentration test for optimal amber suppression in eGFPY40X by pAzF. Wet cell weight was determined at the end of 24 deep-well plate screenings by weighing the pellets from 1 mL culture aliquots. Negative control cultures contained varying amounts of NaOH while the suppression cultures contained varying amounts of pAzF, prepared as a stock solution of 100 mM in 100 mM NaOH. The mean relative cell-associated product was calculated from the ratio of the geometric mean of fluorescence intensity and forward scatter as described by Kolb et al. [1] and Dumas et al. [2]. Average values and the corresponding standard deviations were calculated from three biological replicates. Table S2. List of primers prepared for this study. Table S3. List of strains prepared in this study. [file 12934_2022_1882_MOESM2_ESM.pdf]

From strain engineering to process development: monoclonal antibody production with an unnatural amino acid in *Pichia pastoris*

Nora Tir[1,2], Lina Heistinger[1,2]*, Clemens Grünwald-Gruber[3], Leo A. Jakob[4], Stephan Dickgiesser[5], Nicolas Rasche[5], Diethard Mattanovich[2]**

[1] University of Natural Resources and Life Sciences, Department of Biotechnology, Christian Doppler Laboratory for Innovative Immunotherapeutics, Muthgasse 18, 1190 Vienna, Austria
[2] University of Natural Resources and Life Sciences, Department of Biotechnology, Institute of Microbiology and Microbial Biotechnology, Muthgasse 18, 1190 Vienna, Austria
[3] University of Natural Resources and Life Sciences, Vienna Core Facility Mass Spectrometry Muthgasse 18, 1190 Vienna, Austria
[4] University of Natural Resources and Life Sciences, Department of Biotechnology, Institute of Bioprocess Science and Engineering, Muthgasse 18, 1190 Vienna, Austria
[5] ADCs & Targeted NBE Therapeutics, Merck Healthcare KGaA, Frankfurter Str. 250, 64293 Darmstadt, Germany
* present address: ETH Zürich, Department of Biology, Institute of Biochemistry, 8093 Zürich, Switzerland

**Corresponding author:
Univ. Prof. Dr. Diethard Mattanovich
University of Natural Resources and Life Sciences, Department of Biotechnology, Institute of Microbiology and Microbial Biotechnology
Muthgasse 18, 1190 Vienna, Austria
Email: diethard.mattanovich@boku.ac.at
Phone: +43 1 47654 79006; FAX: +43 1 47654 79009

Table S1. Concentration test for optimal amber suppression in $eGFP_{Y40X}$ by pAzF. Wet cell weight was determined at the end of 24 deep-well plate screenings by weighing the pellets from 1 mL culture aliquots. Negative control cultures contained varying amounts of NaOH while the suppression cultures contained varying amounts of pAzF, prepared as a stock solution of 100 mM in 100 mM NaOH. The mean relative cell-associated product was calculated from the ratio of the geometric mean of fluorescence intensity and forward scatter as described by [1] and [2]. Average values and the corresponding standard deviations were calculated from three biological replicates.

		**Wet cell weight [mg] ± S.D. [mg]**				**Mean relative cell-associated product ± SD**			
	**Concentration [mM]**	eGFP		$eGFP_{Y40X}$ + $tRNA_{CUA}/RS^{pAzF}$		eGFP		$eGFP_{Y40X}$ + $tRNA_{CUA}/RS^{pAzF}$	
**NaOH**	1	71	± 1	71	± 1	90	± 7	0.6	± 0.1
	2	72.4	± 0.3	72	± 1	92	± 8	0.6	± 0.1
	5	73	± 2	72	± 2	92	± 8	0.7	± 0.1
	10	75.4	± 0.5	74	± 2	91	± 8	0.7	± 0.2
**pAzF**	1	64	± 1	63	± 3	99	± 7	36	± 1
	2	58	± 1	58	± 3	102	± 3	41	± 1
	5	52	± 1	56	± 1	100	± 7	39.8	± 0.7
	10	54	± 1	59.2	± 0.6	98	± 4	40.8	± 0.6

References:

1. Hohenblum H, Borth N, Mattanovich D. Assessing Viability and Cell-Associated Product of Recombinant Protein Producing *Pichia Pastoris* with Flow Cytometry. J Biotechnol. 2003;102:281–290.

2. Stadlmayr G, Mecklenbräuker A, Rothmüller M, Maurer M, Sauer M, Mattanovich D, Gasser B. Identification and Characterisation of Novel *Pichia Pastoris* Promoters for Heterologous Protein Production. J Biotechnol. 2010;150:519–529.

Additional file 2

Table S2. List of primers prepared for this study.

Primer name	Sequence (5'->3')
**eGFP_Y40_FS2_fw**	gataGGTCTCCCATGGTGAGCAAGGGC
**eGFP_Y40_FSX_rv**	gataGGTCTCCCTAGGTGGCATCGCC
**eGFP_Y40_FSX_fw**	gataGGTCTCCCTAGGGCAAGCTGACC
**eGFP_Y40_FS3_rv**	gataGGTCTCCAAGCTTACTTGTACAGCTCGTCC
**tRNA_CUA_FS2_fw**	gataGGTCTCCCATGCTCTTTTTCAATTGTATATGTGTTATG
**tRNA_CUA_FSX_rv**	gataGGTCTCCGCTAGCATAAAAAACAAAAAAATGGTGGG
**tRNA_CUA_FSX_fw**	gataGGTCTCCTAGCCTCTTTTTCAATTGTATATGTGTTATGTAG
**tRNA_CUA_FSX´_rv**	gataGGTCTCCGATCGCATAAAAAACAAAAAAATGGTGGG
**tRNA_CUA_FSX´_fw**	gataGGTCTCCGATCCTCTTTTTCAATTGTATATGTGTTATGTAGT
**tRNA_CUA_FS3_rv**	gataGGTCTCCAAGCCATAAAAAACAAAAAAATGGTGGG
**MFalpha_FS2_fw**	gataGGTCTCCCATGAGATTCCCATCTATTTTCAC
**pro-MFalpha_FSX_rv**	gataGGTCTCcTCTTTTCTCGAGAgataCCCCT
**FabHC_FSX_fw**	gagaGGTCTCcaagaGAGGTTCAATTGGTTGAATCTG
**FabHC_FS3_rv**	gataGGTCTCCAAGCTTATTACTTGTCACAGG
**FabLC_FSX_fw**	gagaGGTCTCCaagaGACATTCAGATGACTCAATCTC
**FabLC_FS3_rv**	gagaGGTCTCcAAGCTTATTAACACTCACCTCT
**TraFabLC_Y173_fw**	gagaGGTCTCcTAGTCATTGTCCTCCACTTTGA
**TraFabLC_Y173_rv**	gagaGGTCTCcACTAGGTGGAGTCCTTGG
**TraMabHC_FS3_rv**	gagaGGTCTCcAAGCTCATTATTTACCCGGG
**YAP1_FS2_fw**	gataGGTCTCCCATGAGTGACGTGGTAAACAAG
**YAP1_FS3_rv**	gataGGTCTCCAAGCTTAAACATGGAAAAATCGACAACATC
**CPR5_FS2_fw**	tGAAGACtcCATGAAATTGTTGAACTTTCTGCT
**CPR5_FS3_rv**	tGAAGACtcAAGCTTACAACTCATCTTTCACGACCTC
**SBH1_FS2_fw**	tGAAGACtcCATGTCTACAGCAATTCCAGGAGGAC
**SBH1_FS3_rv**	tGAAGACtcAAGCTTAGTTGATCAACTTTCCTGTCAGCT
**PDI1_FS2_fw**	gataGGTCTCcCATGCAATTCAACTGGAATATTAAAACTGT
**PDI1_FS3_rv**	gataGGTCTCcAAGCTTAAAGCTCGTCGTGA
**HAC1(i)_FS2_fw**	gataGGTCTCcCATGCCCGTAGATTCTTCTCATAA
**HAC1(i)_FS3_rv**	gataGGTCTCcAAGCCTACTATTCCTGGAAGAATAC
**YPT7_KO_gRNA1**	atggtctcCCATGTGGTAGCTGATGAGTCCGTGAGGACGAAACGAGTAAGCTCGTCCTAC
**YPT7_KO_gRNA2**	AAACGAGTAAGCTCGTCCTACCATGGCAGGAGTAGTAgttttagagctagaaatagcaag
**YPT7_KO2_gRNA1**	atggtctcCCATGTAAAATCTGATGAGTCCGTGAGGACGAAACGAGTAAGCTCGTCATTT
**YPT7_KO2_gRNA2**	AAACGAGTAAGCTCGTCATTTTAAAAGTGATTATACTgttttagagctagaaatagcaag
**YPT7_HR1_fw**	GCAgataAAGGAGATGTTACTGAAGG
**oeYPT7_HR1_rv**	TTTCATTAGGGAAGCTGGTGATTTTGCCGGTCTTG
**oeYPT7_HR2_fw**	GGCAAAATCACCAGCTTCCCTAATGAAAGCGGAAATTC
**YPT7_HR2_rv**	CCAGTAATACAACCAGGTGATCC
**YPT7ko_seq_fw**	tGAAGACtcGGAGTAGGGATGGACGAAATATTTGTGAATTC
**YPT7ko_seq_rv**	tGAAGACtcAGCGTATCATTCATGTCAACCAAAGATTGAAG

Table S3. List of strains prepared in this study.

Strain ID	Genotype	Resistance
**CBS2612 + eGFP**	5'-*AOX1*tt-P_{AOX1}_eGFP_*RPS3*tt-*natMX*-*AOX1*tt-3'	NTC
**CBS2612 + eGFP$_{Y40X}$**	5'-*AOX1*tt-P_{AOX1}_eGFP$_{Y40X}$-His$_6$_*RPS3*tt-*natMX*-*AOX1*tt-3'	NTC
**CBS2612 + eGFP$_{Y40X}$ + tRNA$_{CUA}$/RS^{pAzF} (1)**	5'-*AOX1*tt-P_{AOX1}_eGFP$_{Y40X}$-His$_6$_*RPS3*tt-*natMX*-*AOX1*tt-3' 5'-*RGI1*-P_{DAS1}_EcYRS^{pAzF}_*RPS3*tt_P_{DAS2}_3x(*SUP4*-tRNA$_{CUA}$)_*RPS3*tt-*KanMX*-*RGI1*-3'	NTC, G418
**CBS2612 + eGFP$_{Y40X}$ + tRNA$_{CUA}$/RS^{pAzF} (2)**	5'-*AOX1*tt-P_{AOX1}_eGFP$_{Y40X}$-His$_6$_*RPS3*tt-*natMX*-*AOX1*tt-3' 5'-*RGI1*-P_{DAS2}_EcYRS^{pAzF}_*RPS3*tt_P_{DAS1}_3x(*SUP4*-tRNA$_{CUA}$)_*RPS3*tt-*KanMX*-*RGI1*-3'	NTC, G418
**CBS2612 + eGFP$_{Y40X}$ + tRNA$_{CUA}$/RS^{pAzF} (3)**	5'-*AOX1*tt-P_{AOX1}_eGFP$_{Y40X}$-His$_6$_*RPS3*tt-*natMX*-*AOX1*tt-3' 5'-*RGI1*-P_{AOX1}_EcYRS^{pAzF}_*RPS3*tt_P_{DAS2}_3x(*SUP4*-tRNA$_{CUA}$)_*RPS3*tt-*KanMX*-*RGI1*-3'	NTC, G418
**CBS2612 + eGFP$_{Y40X}$ + tRNA$_{CUA}$/RS^{pAzF} (4)**	5'-*AOX1*tt-P_{AOX1}_eGFP$_{Y40X}$-His$_6$_*RPS3*tt-*natMX*-*AOX1*tt-3' 5'-*RGI1*-P_{DAS2}_EcYRS^{pAzF}_*RPS3*tt_P_{AOX1}_3x(*SUP4*-tRNA$_{CUA}$)_*RPS3*tt-*KanMX*-*RGI1*-3'	NTC, G418
**CBS2612 + eGFP$_{Y40X}$ + tRNA$_{CUA}$/RS^{pAzF} (5)**	5'-*AOX1*tt-P_{AOX1}_eGFP$_{Y40X}$-His$_6$_*RPS3*tt-*natMX*-*AOX1*tt-3' 5'-*RGI1*-P_{AOX1}_EcYRS^{pAzF}_*RPS3*tt_P_{AOX1}_3x(*SUP4*-tRNA$_{CUA}$)_*RPS3*tt-*KanMX*-*RGI1*-3'	NTC, G418
**CBS2612 + eGFP$_{Y40X}$ + tRNA$_{CUA}$/RS^{pAzF} (6)**	5'-*AOX1*tt-P_{AOX1}_eGFP$_{Y40X}$-His$_6$_*RPS3*tt-*natMX*-*AOX1*tt-3' 5'-*RGI1*-P_{PMP20}_EcYRS^{pAzF}_*RPS3*tt_P_{AOX1}_3x(*SUP4*-tRNA$_{CUA}$)_*RPS3*tt-*KanMX*-*RGI1*-3'	NTC, G418
**CBS2612 + ppMFα-Fab**	5'-*AOX1*tt-P_{AOX1}_prepro-MFα-TraFabLC_*RPS2*tt_P_{DAS2}_prepro-MFα-TraFabHC_*RPS3*tt-*ZeoR*-*AOX1*tt-3'	Zeo
**CBS2612 + pOpMFα-Fab (i)**	5'-*AOX1*tt-P_{AOX1}_pre-Ost1-pro-MFα-TraFabLC_*RPS2*tt_P_{DAS2}_pre-Ost1-pro-MFα-TraFabHC_*RPS3*tt-*ZeoR*-*AOX1*tt-3'	Zeo

**CBS2612 + pOpMFα-Fab$_{Y173X}$ + tRNA$_{CUA}$/RS^{pAzF} (1)**	5'-*AOX1*tt-P$_{AOX1}$_pre-Ost1-pro-MFα-TraFabLC^{Y173X}_*RPS2*tt_P$_{DAS2}$_pre-Ost1-pro-MFα-TraFabHC_*RPS3*tt-*ZeoR*-*AOX1*tt-3' 5'-*RGI1*-P$_{AOX1}$_EcYRS^{pAzF}_*RPS3*tt_P$_{DAS2}$_3x(*SUP4*-tRNA$_{CUA}$)_*RPS3*tt-*KanMX*-*RGI1*-3'	Zeo, G418
**CBS2612 + pOpMFα-IgG**	5'-*AOX1*tt-P$_{AOX1}$_pre-Ost1-pro-MFα-TraFabLC_*RPS2*tt_P$_{DAS2}$_pre-Ost1-pro-MFα-TraMabHC_*RPS3*tt-*ZeoR*-*AOX1*tt-3'	Zeo
**CBS2612 + pOpMFα-IgG$_{Y173X}$ + tRNA$_{CUA}$/RS^{pAzF} (1)**	5'-*AOX1*tt-P$_{AOX1}$_pre-Ost1-pro-MFα-TraFabLC^{Y173X}_*RPS2*tt_P$_{DAS2}$_pre-Ost1-pro-MFα-TraMabHC_*RPS3*tt-*ZeoR*-*AOX1*tt-3' 5'-*RGI1*-P$_{AOX1}$_EcYRS^{pAzF}_*RPS3*tt_P$_{DAS2}$_3x(*SUP4*-tRNA$_{CUA}$)_*RPS3*tt-*KanMX*-*RGI1*-3'	Zeo, G418
**CBS2612 + pOpMFα-Fab (ii)**	5'-*AOX1*tt-P$_{AOX1}$_pre-Ost1-pro-MFα-TraFabLC_*RPS2*tt_P$_{AOX1}$_pre-Ost1-pro-MFα-TraFabHC_*RPS3*tt-*ZeoR*-*AOX1*tt-3'	Zeo
**CBS2612 + pOpMFα-Fab (iii)**	5'-*AOX1*tt-P$_{AOX1}$_pre-Ost1-pro-MFα-TraFabLC_*RPS2*tt_P$_{DAS1}$_pre-Ost1-pro-MFα-TraFabHC_*RPS3*tt-*ZeoR*-*AOX1*tt-3'	Zeo
**CBS2612 + pOpMFα-Fab (iv)**	5'-*AOX1*tt-P$_{DAS1}$_pre-Ost1-pro-MFα-TraFabLC_*RPS2*tt_P$_{AOX1}$_pre-Ost1-pro-MFα-TraFabHC_*RPS3*tt-*ZeoR*-*AOX1*tt-3'	Zeo
**CBS2612 + pOpMFα-Fab (v)**	5'-*AOX1*tt-P$_{PMP20}$_pre-Ost1-pro-MFα-TraFabLC_*RPS2*tt_P$_{AOX1}$_pre-Ost1-pro-MFα-TraFabHC_*RPS3*tt-*ZeoR*-*AOX1*tt-3'	Zeo
**CBS2612 + pOpMFα-Fab (vi)**	5'-*AOX1*tt-P$_{DAS2}$_pre-Ost1-pro-MFα-TraFabLC_*RPS2*tt_P$_{AOX1}$_pre-Ost1-pro-MFα-TraFabHC_*RPS3*tt-*ZeoR*-*AOX1*tt-3'	Zeo
**CBS2612 + pOpMFα-IgG + *YAP1*oe**	5'-*AOX1*tt-P$_{AOX1}$_pre-Ost1-pro-MFα-TraFabLC_*RPS2*tt_P$_{DAS2}$_pre-Ost1-pro-MFα-TraMabHC_*RPS3*tt-*ZeoR*-*AOX1*tt-3' 5'-*ENO1*-P$_{GAP}$_*YAP1*_*RPS3*tt-*HphMX*-*ENO1*-3'	Zeo, Hyg
**CBS2612 + pOpMFα-IgG + *HAC1*(i)oe**	5'-*AOX1*tt-P$_{AOX1}$_pre-Ost1-pro-MFα-TraFabLC_*RPS2*tt_P$_{DAS2}$_pre-Ost1-pro-MFα-TraMabHC_*RPS3*tt-*ZeoR*-*AOX1*tt-3' 5'-*ENO1*-P$_{GAP}$_*HAC1*(i)_*RPS3*tt-*HphMX*-*ENO1*-3'	Zeo, Hyg
**CBS2612 + pOpMFα-IgG + *PDI1*oe**	5'-*AOX1*tt-P$_{AOX1}$_pre-Ost1-pro-MFα-TraFabLC_*RPS2*tt_P$_{DAS2}$_pre-Ost1-pro-MFα-TraMabHC_*RPS3*tt-*ZeoR*-*AOX1*tt-3' 5'-*ENO1*-P$_{GAP}$_*PDI1*_*RPS3*tt-*HphMX*-*ENO1*-3'	Zeo, Hyg

**CBS2612 + pOpMFα-IgG + *CPR5*oe**	5'-*AOX1*tt-P_{AOX1}_pre-Ost1-pro-MFα-TraFabLC_*RPS2*tt_P_{DAS2}_pre-Ost1-pro-MFα-TraMabHC_*RPS3*tt-*ZeoR*-*AOX1*tt-3' 5'-*ENO1*-P_{TEF}_*CPR5*_*RPS2*tt-*HphMX*-*ENO1*-3'	Zeo, Hyg
**CBS2612 + pOpMFα-IgG + *SBH1*oe**	5'-*AOX1*tt-P_{AOX1}_pre-Ost1-pro-MFα-TraFabLC_*RPS2*tt_P_{DAS2}_pre-Ost1-pro-MFα-TraMabHC_*RPS3*tt-*ZeoR*-*AOX1*tt-3' 5'-*ENO1*-P_{TEF}_*SBH1*_*RPS2*tt-*HphMX*-*ENO1*-3'	Zeo, Hyg
**CBS2612 + pOpMFα-IgG + *KAR2*oe**	5'-*AOX1*tt-P_{AOX1}_pre-Ost1-pro-MFα-TraFabLC_*RPS2*tt_P_{DAS2}_pre-Ost1-pro-MFα-TraMabHC_*RPS3*tt-*ZeoR*-*AOX1*tt-3' 5'-*ENO1*-P_{GAP}_*KAR2*_*RPS3*tt-*HphMX*-*ENO1*-3'	Zeo, Hyg
**CBS2612 + pOpMFα-IgG + *LHS1*oe**	5'-*AOX1*tt-P_{AOX1}_pre-Ost1-pro-MFα-TraFabLC_*RPS2*tt_P_{DAS2}_pre-Ost1-pro-MFα-TraMabHC_*RPS3*tt-*ZeoR*-*AOX1*tt-3' 5'-*ENO1*-P_{POR1}_*LHS1*_*IDP1*tt-*HphMX*-*ENO1*-3'	Zeo, Hyg
**CBS2612 + pOpMFα-IgG + *KAR2*+*LHS1*oe**	5'-*AOX1*tt-P_{AOX1}_pre-Ost1-pro-MFα-TraFabLC_*RPS2*tt_P_{DAS2}_pre-Ost1-pro-MFα-TraMabHC_*RPS3*tt-*ZeoR*-*AOX1*tt-3' 5'-*ENO1*-P_{GAP}_*KAR2*_*RPS3*tt_P_{POR1}_*LHS1*_*IDP1*tt-*HphMX*-*ENO1*-3'	Zeo, Hyg
**CBS2612_Δ*YPT7* + pOpMFα-Fab**	Δ*ypt7* 5'-*AOX1*tt-P_{AOX1}_pre-Ost1-pro-MFα-TraFabLC_*RPS2*tt_P_{DAS2}_pre-Ost1-pro-MFα-TraFabHC_*RPS3*tt-*ZeoR*-*AOX1*tt-3'	Zeo
**CBS2612_Δ*YPT7* + pOpMFα-Fab_{Y173X} + $tRNA_{CUA}$/RS^{pAzF} (1)**	Δ*ypt7* 5'-*AOX1*tt-P_{AOX1}_pre-Ost1-pro-MFα-TraFabLC^{Y173X}_*RPS2*tt_P_{DAS2}_pre-Ost1-pro-MFα-TraFabHC_*RPS3*tt-*ZeoR*-*AOX1*tt-3' 5'-*RGI1*-P_{AOX1}_EcYRS^{pAzF}_*RPS3*tt_P_{DAS2}_3x(*SUP4*-$tRNA_{CUA}$)_*RPS3*tt-*KanMX*-*RGI1*-3'	Zeo, G418
**CBS2612_Δ*YPT7* + pOpMFα-IgG**	Δ*ypt7* 5'-*AOX1*tt-P_{AOX1}_pre-Ost1-pro-MFα-TraFabLC_*RPS2*tt_P_{DAS2}_pre-Ost1-pro-MFα-TraMabHC_*RPS3*tt-*ZeoR*-*AOX1*tt-3'	Zeo
**CBS2612_Δ*YPT7* + pOpMFα-IgG_{Y173X} + $tRNA_{CUA}$/RS^{pAzF} (1)**	Δ*ypt7* 5'-*AOX1*tt-P_{AOX1}_pre-Ost1-pro-MFα-TraFabLC^{Y173X}_*RPS2*tt_P_{DAS2}_pre-Ost1-pro-MFα-TraMabHC_*RPS3*tt-*ZeoR*-*AOX1*tt-3' 5'-*RGI1*-P_{AOX1}_EcYRS^{pAzF}_*RPS3*tt_P_{DAS2}_3x(*SUP4*-$tRNA_{CUA}$)_*RPS3*tt-*KanMX*-*RGI1*-3'	Zeo, G418

**CBS2612_Δ*YPT7* + pOpMFα-Fab + *KAR2*+*LHS1*oe**	Δ*ypt7* 5'-*AOX1*tt-P_{AOX1}_pre-Ost1-pro-MFα-TraFabLC_*RPS2*tt_P_{DAS2}_pre-Ost1-pro-MFα-TraFabHC_*RPS3*tt-*ZeoR*-*AOX1*tt-3' 5'-*ENO1*-P_{GAP}_*KAR2*_*RPS3*tt_P_{POR1}_*LHS1*_*IDP1*tt-*HphMX*-*ENO1*-3'	Zeo, Hyg
**CBS2612_Δ*YPT7* + pOpMFα-Fab$_{Y173X}$ + tRNA$_{CUA}$/RS^{pAzF} (1) + *KAR2*+*LHS1*oe**	Δ*ypt7* 5'-*AOX1*tt-P_{AOX1}_pre-Ost1-pro-MFα-TraFabLC^{Y173X}_*RPS2*tt_P_{DAS2}_pre-Ost1-pro-MFα-TraFabHC_*RPS3*tt-*ZeoR*-*AOX1*tt-3' 5'-*RGI1*-P_{AOX1}_EcYRS^{pAzF}_*RPS3*tt_P_{DAS2}_3x(*SUP4*-tRNA$_{CUA}$)_*RPS3*tt-*KanMX*-*RGI1*-3' 5'-*ENO1*-P_{GAP}_*KAR2*_*RPS3*tt_P_{POR1}_*LHS1*_*IDP1*tt-*HphMX*-*ENO1*-3'	Zeo, G418, Hyg
**CBS2612_Δ*YPT7* + pOpMFα-IgG + *KAR2*+*LHS1*oe**	Δ*ypt7* 5'-*AOX1*tt-P_{AOX1}_pre-Ost1-pro-MFα-TraFabLC_*RPS2*tt_P_{DAS2}_pre-Ost1-pro-MFα-TraMabHC_*RPS3*tt-*ZeoR*-*AOX1*tt-3' 5'-*ENO1*-P_{GAP}_*KAR2*_*RPS3*tt_P_{POR1}_*LHS1*_*IDP1*tt-*HphMX*-*ENO1*-3'	Zeo, Hyg
**CBS2612_Δ*YPT7* + pOpMFα-IgG$_{Y173X}$ + tRNA$_{CUA}$/RS^{pAzF} (1) + *KAR2*+*LHS1*oe**	Δ*ypt7* 5'-*AOX1*tt-P_{AOX1}_pre-Ost1-pro-MFα-TraFabLC^{Y173X}_*RPS2*tt_P_{DAS2}_pre-Ost1-pro-MFα-TraMabHC_*RPS3*tt-*ZeoR*-*AOX1*tt-3' 5'-*RGI1*-P_{AOX1}_EcYRS^{pAzF}_*RPS3*tt_P_{DAS2}_3x(*SUP4*-tRNA$_{CUA}$)_*RPS3*tt-*KanMX*-*RGI1*-3' 5'-*ENO1*-P_{GAP}_*KAR2*_*RPS3*tt_P_{POR1}_*LHS1*_*IDP1*tt-*HphMX*-*ENO1*-3'	Zeo, G418, Hyg
